# Lactate dehydrogenases amplify reactive oxygen species in cancer cells in response to oxidative stimuli

**DOI:** 10.1038/s41392-021-00595-3

**Published:** 2021-06-28

**Authors:** Hao Wu, Yuqi Wang, Minfeng Ying, Chengmeng Jin, Jiangtao Li, Xun Hu

**Affiliations:** 1 Cancer Institute (Key Laboratory for Cancer Intervention and Prevention, China National Ministry of Education, Zhejiang Provincial Key Laboratory of Molecular Biology in Medical Sciences), The Second Affiliated Hospital, Zhejiang University School of Medicine, Hangzhou, China; 2 Department of Surgery, The Second Affiliated Hospital, Zhejiang University School of Medicine, Hangzhou, China

**Keywords:** Cancer metabolism, Cancer metabolism

## Abstract

Previous studies demonstrated that superoxide could initiate and amplify LDH-catalyzed hydrogen peroxide production in aqueous phase, but its physiological relevance is unknown. Here we showed that LDHA and LDHB both exhibited hydrogen peroxide-producing activity, which was significantly enhanced by the superoxide generated from the isolated mitochondria from HeLa cells and patients’ cholangiocarcinoma specimen. After LDHA or LDHB were knocked out, hydrogen peroxide produced by Hela or 4T1 cancer cells were significantly reduced. Re-expression of LDHA in LDHA-knockout HeLa cells partially restored hydrogen peroxide production. In HeLa and 4T1 cells, LDHA or LDHB knockout or LDH inhibitor FX11 significantly decreased ROS induction by modulators of the mitochondrial electron transfer chain (antimycin, oligomycin, rotenone), hypoxia, and pharmacological ROS inducers piperlogumine (PL) and phenethyl isothiocyanate (PEITC). Moreover, the tumors formed by LDHA or LDHB knockout HeLa or 4T1 cells exhibited a significantly less oxidative state than those formed by control cells. Collectively, we provide a mechanistic understanding of a link between LDH and cellular hydrogen peroxide production or oxidative stress in cancer cells in vitro and in vivo.

## Introduction

Hydrogen peroxide (H_2_O_2_) plays important roles in cancer initiation and development,^1,2^ but the molecular mechanism underlying H_2_O_2_ production in cancer cells is not completely understood. According to current understanding, cellular H_2_O_2_ is mainly produced by superoxide dismutase (SOD). However, LDH may also contribute to cellular H_2_O_2_. Previously, Chan and Bielski found that rabbit LDH could catalyze one-electron reduction of NADH to produce H_2_O_2_, with the superoxide as an initiator of the reaction.^3,4^ Later, Patrat et al. demonstrated that molecular oxygen, H_2_O_2_, peroxynitrite, superoxide all could initiate this chain of free radical reaction on rabbit LDH-bounded NADH,^5^ but only superoxide can amplify the reactions, in the following sequential reactions:

1 $${\mathrm{LDH}} - {\mathrm{NADH}} + {\mathrm{H}}^ + + {\mathrm{O}}_2^{ \bullet - } \to {\mathrm{LDH}} - {\mathrm{NAD}}^ \bullet + {\mathrm{H}}_2{\mathrm{O}}_2$$

2 $${\mathrm{LDH}} - {\mathrm{NAD}}^ \bullet + {\mathrm{O}}_2 \to {\mathrm{LDH}} - {\mathrm{NAD}}^ + + {\mathrm{O}}_2^{ \bullet - }$$

3 $${\mathrm{LDH}} - {\mathrm{NAD}}^ + + {\mathrm{NADH}} \to {\mathrm{LDH}} - {\mathrm{NADH}} + {\mathrm{NAD}}^ +$$

4 $${\mathrm{LDH}} - {\mathrm{NADH}} + {\mathrm{H}}^ + + {\mathrm{O}}_2^{ \bullet - } \to {\mathrm{LDH}} - {\mathrm{NAD}}^ \bullet + {\mathrm{H}}_2{\mathrm{O}}_2$$

In one cycle of the reactions, one molecule of H_2_O_2_ is generated and the molecule of superoxide is recycled hence it can initiate another cycle of the above reaction. As a result, one molecule of superoxide can initiate many round reactions, leading to generation of many molecules of H_2_O_2_. Although these studies build solid theoretical basis for LDH-involved H_2_O_2_ production, it has not attracted much attention since then. Up to date, it is unclear if LDH-catalyzed production of H_2_O_2_ is physiologically relevant. In theory, LDH-catalyzed H_2_O_2_ production should also present in cancer cells, on the following basis. First, superoxide radical, the initiator and amplifier, can be generated from various sources. It is estimated that about 0.12–2% of respiration in the mitochondria is converted to superoxide in vitro, and less in vivo.^6^ Electron leak from the complex III and mitochondrial glycerol-3-phosphate dehydrogenase (mGPDH) on the inner membrane of mitochondria could be captured by molecular oxygen to form superoxide, which can be released toward the intermembrane space or cytosol side.^7,8^ The mitochondrial outer membrane is permeable to small molecules with MW less than 5000 dalton, hence superoxide could diffuse from intermembrane space to cytosol.^9^ The other sources of superoxide generation in cells include NAPDH oxidase, xanthine oxidase, cytochrome P450 peroxidases.^10,11^ When tetrahydrobiopterin and arginine is low, nitric oxide synthase also generate superoxide.^12^ Second, most cancer cells exhibit very high glycolysis rate, termed Warburg effect,^13^ which can efficiently cycle NAD^+^ to NADH, which is a substrate to maintain the free radical chain reactions catalyzed by LDH. Finally, increased level of LDHA is characteristic of many tumors.^14–16^ LDH activities in cultured cancer cell lines are very high, e.g., LDH activities in cervical cancer cell line HeLa, gastric cancer cell line MGC803, colon cancer cell line RKO, lung cancer cell line A549, and liver cancer cell line SK-HEP-1 are between 4521.1 and 7613.1 nmol/(min.mg protein).^17^ Based on the abundant sources of superoxide, adequate flux of NADH generation, and very high activity of LDH in cancer cells, we propose that LDH may contribute significantly to H_2_O_2_ production and oxidative stress in cancer cells in vitro and in vivo.

## Results

### H_2_O_2_-generation activity of LDH

**Fig. 1:**
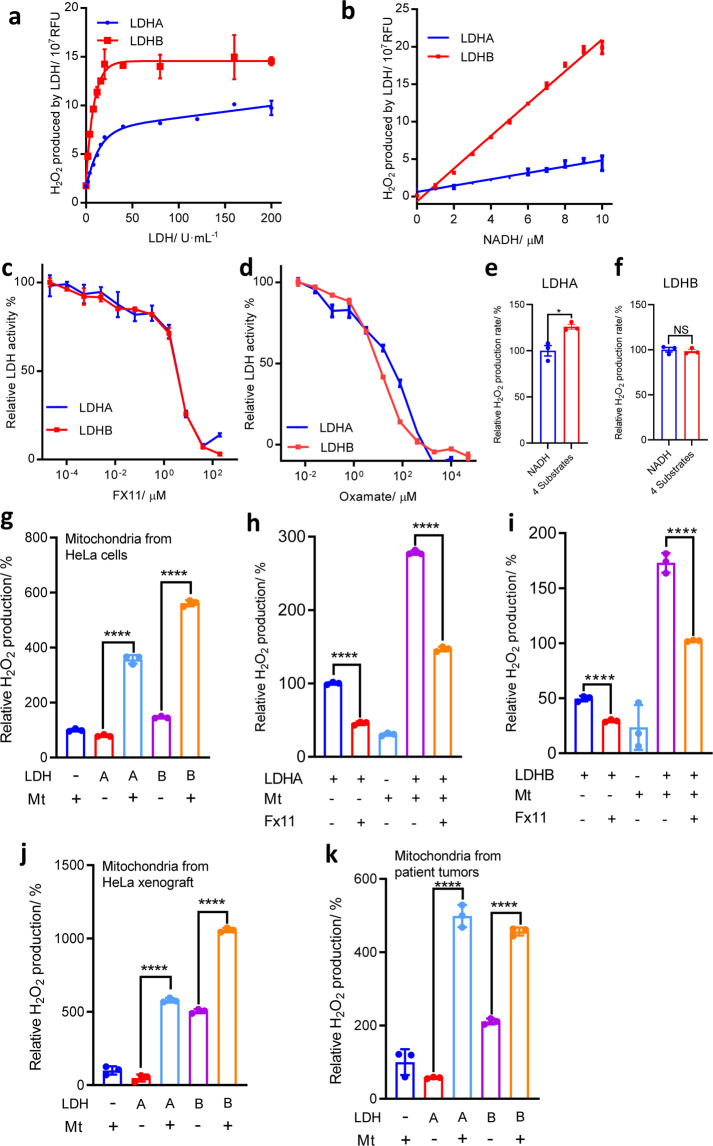
The H_2_O_2_-generating activity of LDHA and LDHB. Purified recombinant LDHA and LDHB were incubated with NADH and the H_2_O_2_ generation was detected by Amplex Red-HRP system, as described in Methods. Substrates, inhibitors, and/or mitochondria from different sources were added together as indicated. LDHA and LDHB were assayed under the same condition. **a** LDH concentration-dependent H_2_O_2_ generation. 1 unit of LDH refers the conversion of 1 µmole of pyruvate into lactate in 1 min. **b** NADH concentration-dependent H_2_O_2_ generation by LDHs. **c** Inhibition of H_2_O_2_-generating activity of LDHA and LDHB by FX11. **d** inhibition of H_2_O_2_-generating activity of LDHA and LDHB by oxamate. **e**, **f** LDH-catalyzed generation of H_2_O_2_ with four substrates (pyruvate, lactate, NADH, and NAD) at equilibrium state in the reaction system. The concentrations of pyruvate, lactate, NADH, and NAD were 0.1 mM, 20 mM, 5 μM, 200 μM, so that the reaction quotient is 2 × 10^11^ M^−1^, approximately equal to the equilibrium constant reported.^18,19^ **g** LDH H_2_O_2_-producing activity enhanced by the HeLa cell mitochondria-derived superoxide/ROS increases. Succinate as the electron donor for ETC (electron transfer chain). **h** FX11 inhibited H_2_O_2_ generation in mitochondria-LDHA co-incubation system. **i** FX11 inhibited H_2_O_2_ generation in mitochondria-LDHB co-incubation system. **j**, **k** LDH H_2_O_2_-producing activity enhanced by the mitochondria-derived superoxide/ROS increases. The reaction system contains mitochondria freshly isolated from HeLa tumor xenograft (**j**), or surgically-resected cholangiocarcinoma samples from patients (**k**), succinate as the electron donor for ETC of mitochondria, rotenone, NADH, and LDHA or LDHB, then the H_2_O_2_-producing rate was recorded. Experiments were repeated two times for patients’ samples and three times for others and one representative data is shown and expressed as mean ± SD. RFU relative fluorescence units, Mt mitochondria

The difference of the catalytic rate of LDHA and LDHB in H_2_O_2_ production is unknown. We used purified recombinant human LDHA and LDHB for the assay. LDHB per unit wise displayed significantly higher activity than LDHA with respect to H_2_O_2_ generating (Fig. 1a, b, Supplementary Fig. 1a, b), although they showed similar activities in terms of pyruvate to lactate conversion (Supplementary Fig. 1e).

H_2_O_2_ generation rate was dependent on the concentration of LDH enzymes (Fig. 1a) and the concentration of NADH (Fig. 1b). The signal saturated at high enzyme concentration is probably due to the limited concentration of superoxide in the reaction system (Fig. 1a). These results agreed with the previous reports, that LDH catalyzed a chain of free radical reactions, which was initiated by superoxide in aqueous phase^3^ (Eqs. 1–4). The H_2_O_2_ generation was inhibited by LDH inhibitor FX11 (NAD binding site) (Fig. 1c) and oxamate (pyruvate binding site) (Fig. 1d), with the former being a significantly stronger inhibitor than the latter, suggesting that the enzyme catalytic core was necessary to H_2_O_2_ generation activity.

In the above assay, only one substrate (NADH) was present, but in living cancer cells, four substrates of LDH are present at near-equilibrium state for the primary reactions (pyruvate to lactate).^17^ The equilibrium constant of this reaction is 1.62 × 10^11^ M^−1^ ^18^ or 4 × 10^11^ M^−1^.^19^ In our previous studies, we found that in cells, when lactate and pyruvate concentrations were around 20 and 0.1 mM, the reaction is at near equilibrium,^20–22^ while the concentrations of cytosolic free NAD^+^ and NADH were not able to be measured. In the assay system for measuring H_2_O_2_ production by LDH, the optimal concentration of NADH is 5 μM, above which NADH would introduce a high background fluorescence to interfere the measurement. Hence, we set the reaction mixture that contains 20 mM lactate, 0.1 mM pyruvate, 5 μM NADH, 200 μM NAD^+^, that yields a reaction quotient of 2 × 10^11^ M^−1^. Under this situation, LDH carry out the hydride ion transfer from NADH to pyruvate or in reverse, so that one-electron transfer from NDAH to molecular oxygen may be inhibited. However, when all four substrates presented in the reaction system at near-equilibrium state, LDH produced moderately higher (LDHA) or similar (LDHB) amount of H_2_O_2_ as compared with LDH in the presence of NADH alone (Fig. 1e–f). This experiment indicated that when LDH was assayed with 4 substrates, the H_2_O_2_ generating activity was not inhibited.

We further tested another pair of commercially available LDHs, bovine LDHA and LDHB, which showed the same pattern as human ones (Supplementary Fig. 1a–d).

Mitochondrion is the most prominent superoxide generator in many mammalian cells. Superoxide could be released into both sides of the inner membrane of mitochondria. It has been confirmed that electron leaked from complex III could be released into the intermembrane space,^6,7,23^ where the electron is captured by molecular oxygen to form superoxide radical. In the glycerol 3-phosphate shuttle, glycerol 3-phosphate in the intermembrane space is dehydrogenated by mGPDH, which transfers electron to quinone in the inner membrane of mitochondria then to complex III. During electron transferring from G3P (glycerol 3-phosphate) to quinone, the electron could leak toward the intermembrane space, where it is captured by molecular oxygen to form superoxide radical.^24^ Therefore, mitochondria-produced ROS could serve as initiator for cytosolic LDH to produce H_2_O_2_. We purified functional mitochondria from HeLa cell (Supplementary Fig. 2a). According to the well-established method,^25,26^ in a reaction system containing mitochondria, rotenone, and succinate as the electron donor, a fraction of electron would leak from ETC and captured by molecular oxygen to form superoxide. While the superoxide released into the mitochondrial matrix side is converted to H_2_O_2_ by intramitochondrial SOD, the superoxide released to intermembrane side may function as the initiator for LDH to generate H_2_O_2_. We added LDH and NADH into this reaction system, and observed a significant increase of H_2_O_2_, which could be inhibited by FX11 (Fig. 1g–i). The same held true for mitochondria prepared from HeLa cell xenograft (Fig. 1j and Supplementary Fig. 2b, c). We then prepared functional mitochondria from surgically-resected cholangiocarcinoma samples from 2 patients (Supplementary Fig. 2d) and got the same results (Fig. 1k). By using G3P to displace succinate in the reaction system, we also observed that LDH significantly enhanced the production of H_2_O_2_ (Supplementary Fig. 2e).

Mitochondria from cultured HeLa cell line significantly increased the H_2_O_2_-generation activity of rabbit muscle LDH (Supplementary Fig. 2f). We also got similar results by mixing mitochondria prepared from mouse muscle (Supplementary Fig. 3a) with either commercial LDH from rabbit muscle (Supplementary Fig. 3b) or human recombinant LDH (Supplementary Fig. 3c, d).

Collectively, the data demonstrated that superoxide generated by mitochondria could initiate LDH to generate H_2_O_2_.

### The relevance of LDH with H_2_O_2_ production by cells

**Fig. 2:**
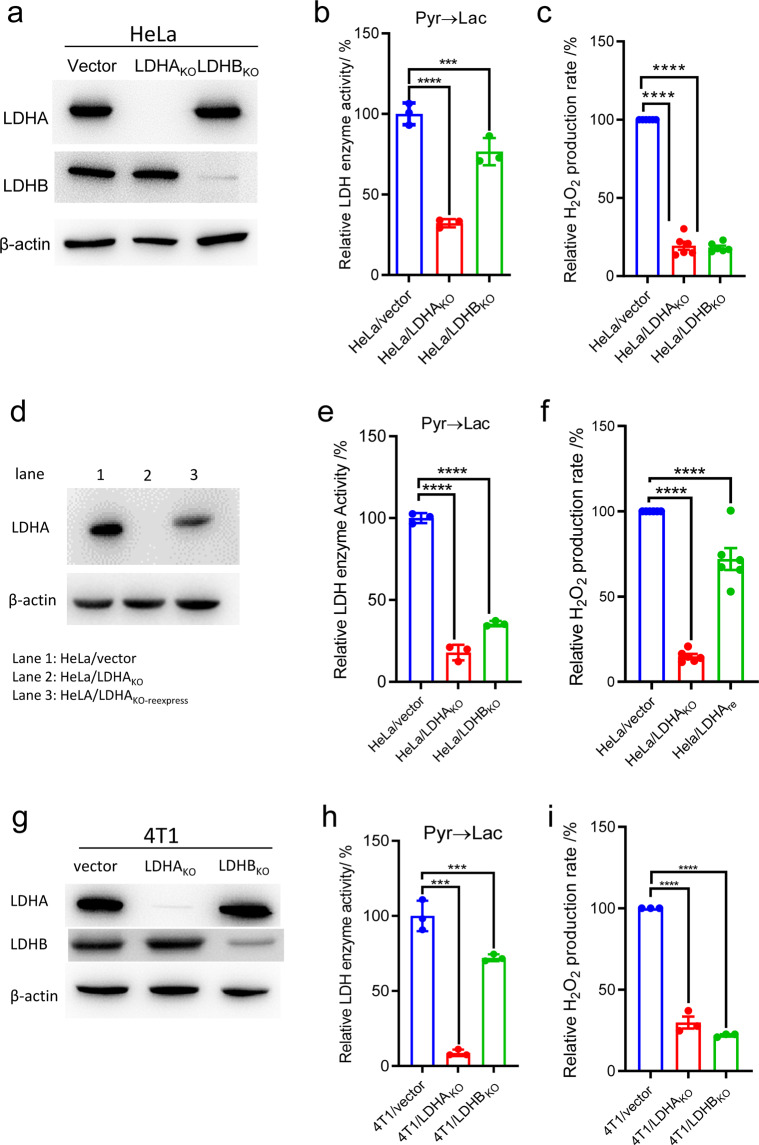
The western blot, LDH enzyme activity and H_2_O_2_ production rate in HeLa and 4T1 cells. **a** Western blot confirmation of LDH knockout in HeLa LDH knockout cell lines. **b** Specific LDH enzyme activity assayed by converting pyruvate to lactate in HeLa LDH knockout cells. **c** Relative H_2_O_2_ production rate in HeLa LDH knockout cells. **d** Western blot confirmation of LDH re-expression in HeLa LDH knockout cell lines. **e** Specific LDH enzyme activity assayed by converting pyruvate to lactate in HeLa LDH knockout and re-expression cells. **f** Relative H_2_O_2_ production rate in HeLa LDH knockout and re-expression cells. **g** Western blot confirmation of LDH knockout in 4T1 LDH knockout cell lines. **h** Specific LDH enzyme activity assayed by converting pyruvate to lactate in 4T1 LDH knockout cells. **i** Relative H_2_O_2_ production rate in 4T1 LDH knockout cells. Data were confirmed by at least three independent experiments. The experimental details are described in Materials and Methods

The next question is if LDH is relevant with H_2_O_2_ production in cells. We established LDHA or LDHB knockout HeLa cell lines. LDHA or LDHB knockout was confirmed by DNA sequencing (Supplementary Fig. 4a), western blot of LDH protein (Fig. 2a), and the specific enzyme activity of LDH (Fig. 2b). In comparison to control HeLa cells, LDHA or LDHB knockout reduced H_2_O_2_ production significantly (Fig. 2c). Re-expression of LDHA in Hela/LDHA_KO_ cells (Fig. 2d, e) partially restored the H_2_O_2_ production (Fig. 2f). The partial recovery of H_2_O_2_ production by Hela/LDHA_re_ was due to that LDH re-expression only partially recovered the LDH activity (Fig. 2d–f). Similar results were reproduced by using LDHA or B knockout 4T1 cells (Supplementary Fig. 4b, Fig. 2g–i).

### The antioxidative and pro-oxidative activity of LDH in cancer cells

The effect of LDH knockout on cellular H_2_O_2_ production indicates that LDH functions as a prooxidant. On the other hand, the effect of perturbation of LDH on oxidative stress is a more complicated issue, when total ROS is concerned, as H_2_O_2_ only represents one species of numerous ROS. Previous reports demonstrated that LDH-inhibition enhances cellular ROS production measured by ROS probes DCFH and MitoSOX RED,^27,28^ indicating that LDH functions as an antioxidant. The authors^27,28^ interpreted that LDH inhibition redirects pyruvate to mitochondrial metabolism thereby enhancing ROS production, but the underlying mechanism remains unknown. Since the antioxidative activity of LDH relies on mitochondria, this activity of LDH should be exhibited in mitochondria intact cells but not mitochondria defective cells (ρ0 cells). If this logic is correct, the antioxidative and pro-oxidative functions of LDH in living cells could be dissected.

**Fig. 3:**
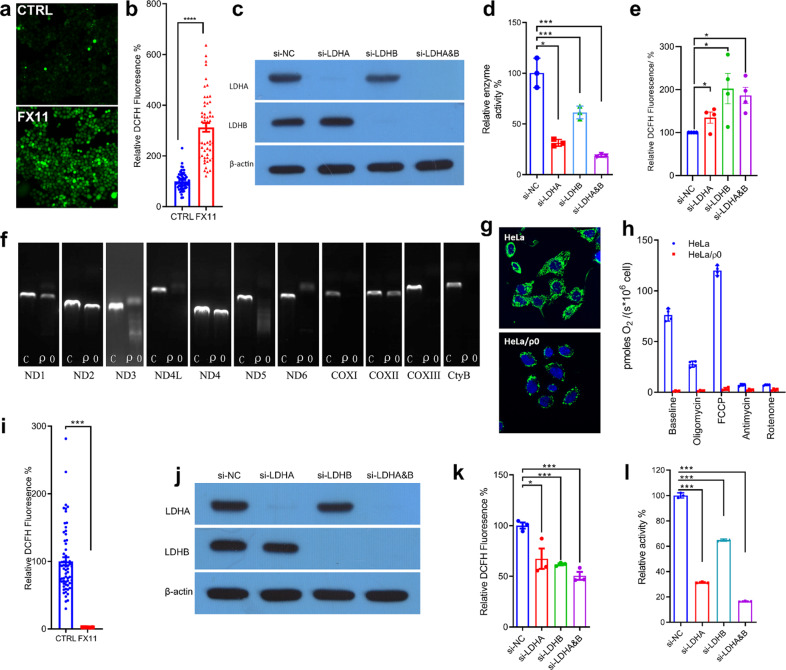
The antioxidative activity of LDH in HeLa cells and the pro-oxidative activity of LDH in HeLa/ρ0 cells. **a**, **b** LDH inhibitor FX11 increases total cellular ROS level. HeLa cells were treated with or without 15 µM FX11 for 30 min and loaded with 10 µM DCFH-DA. Fluorescence signal was recorded by microscope imaging (**a**) and total signal of each cells was calculated for analysis (**b**). **c** Western Blot of LDHA and LDHB of the cell lysate prepared from siRNA transfected HeLa cells. HeLa/si-NC, negative control; HeLa/si-LDHA, LDHA knockdown cell; HeLa/si-LDHB cells, LDHB knockdown cell; HeLa/si-LDHA&B, LDHA and LDHB double knockdown cell. **d** Specific enzyme activity of the cell lysate prepared from HeLa/si-NC, HeLa/si-LDHA, HeLa/si-LDHB, and HeLa/si-LDHA&B cells. The specific enzyme activity refers the one that converts pyruvate to lactate. **e** The total cellular ROS level probed by DCFH in HeLa/si-NC, HeLa/si-LDHA, HeLa/si-LDHB, and HeLa/si-LDHA&B cells. **f** Electrophoresis of PCR products showed HeLa/ρ0 cells lacked mitochondrial DNA (mtDNA)-coded genes, which are essential for ETC complexes. **g** HeLa/ρ0 cells had less mitochondria mass compared with parental HeLa cell. Cells were stained with MitoTraker Green, a Mitochondrion-selective probe, and imaged under confocal microscope. **h** Oxygen consumption rate (OCR) in HeLa cells and HeLa/ρ0 cells. **i** LDH inhibitor FX11 reduces total ROS level in mitochondrial DNA deficient HeLa/ρ0 cells, probed by DCFH. **j** Western blot confirmation of LDHA or LDHB knockdown in HeLa/ρ0 cells by siRNA. **k** Knockdown of LDH isoforms significantly decreases the total cellular ROS level in HeLa/ρ0 cells. **l** LDH knockdown decreased specific activity of LDH in HeLa/ρ0 cells. Data were confirmed by at least three independent experiments. The experimental details are described in Materials and Methods

In HeLa cells, inhibition of LDH activity by small molecule inhibitor FX11 or by siRNA knockdown induced a significant increase of total cellular ROS measured by DCFH probe (Fig. 3a–e).

FX11 increased mitochondrial ROS in HeLa cells (Supplementary Fig. 5). EUK134, a mitochondrial ROS scavenger, inhibited FX11-induced ROS increase in mitochondria and cytosol (Supplementary Fig. 6a). mitoTEMPO, another mitochondrial ROS scavenger, also inhibited FX11-induced ROS increase but with a much less efficacy than EUK134 (Supplementary Fig. 6b).

We then tested the effect of inhibiting LDH in HeLa/ρ0 cells on cellular ROS production. HeLa/ρ0 cells were mitochondrial DNA deficient and lacked essential components of ETC complex coded by mtDNA (Fig. 3f). They had much less mitochondria mass (Fig. 3g) and negligible OXPHOS activity (Fig. 3h), in comparison to HeLa cells. The basal levels of cellular ROS and mitochondrial ROS/superoxide in HeLa/ρ0 cells were significantly lower than those in HeLa cells (Supplementary Fig. 7a–d), the same held true for HCT116/ρ0 cell (Supplementary Fig. 7e–j). As expected, inhibition of LDH by FX11 did not induce an oxidative stress, instead, it decreased total cellular ROS in HeLa/ρ0 cells (Fig. 3i). LDHA or LDHB or both knockdown (Fig. 3j, l) also led to a reduction of cellular ROS in HeLa/ρ0 cells (Fig. 3k).

Taken together, by using ρ0 cells, we demonstrated the pro-oxidative activity of LDHA and LDHB; using wild-type cells, we observed that inhibition of LDH induced an oxidative stress. Thus, we dissected the pro-oxidative and antioxidative activity of LDH in cancer cells.

### The relationship between mitochondrial ROS/superoxide and LDH-mediated cellular ROS

Next, we sought to test if there is a quantitative relationship between mitochondrial ROS/superoxide and LDH-mediated cellular ROS. We treated cells with rotenone, antimycin, or oligomycin. Rotenone inhibits electron transfer from complex I to ubiquinone hence would saturate complex I with electron, eventually leading to electron leakage and producing superoxide.^29^ Antimycin inhibits electron transfer from complex III to ubiquinone, leading to electron saturation at complex I, complex II, and complex III, eventually increasing electron leakage.^30^ Oligomycin A is an inhibitor of F_o_ part of ATP synthase.^31^

**Fig. 4:**
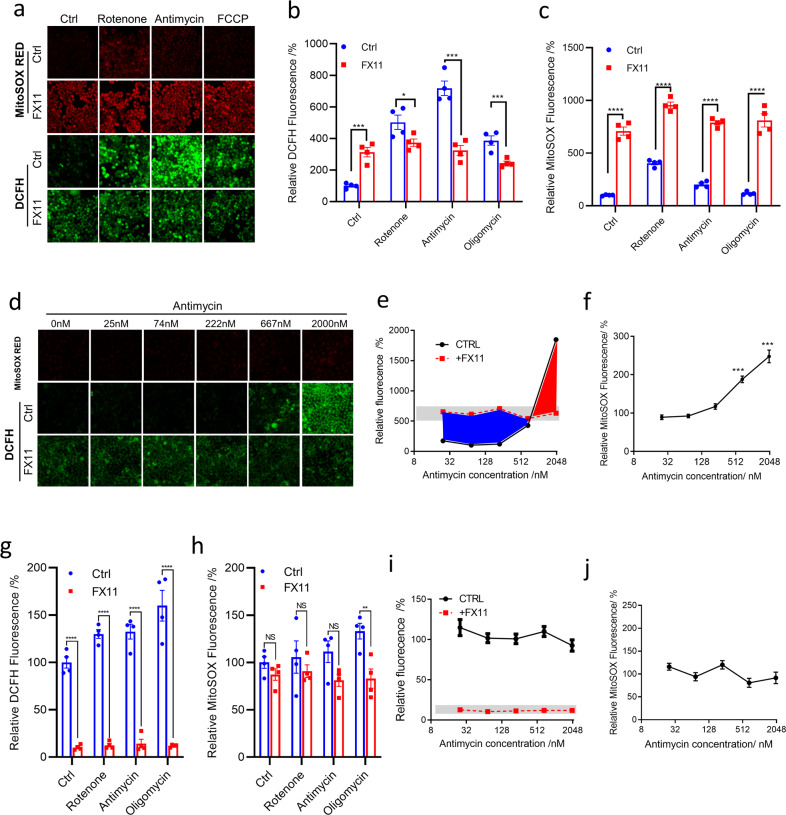
Demonstration of dominant antioxidative and dominant pro-oxidative activity of LDH in HeLa and HeLa/ρ0 cells. **a**–**d** HeLa cells; e-g, HeLa/ρ0 cells). **a** Rotenone-, antimycin-, or oligomycin-induced production of mitochondrial ROS/superoxide (MitoSOX™ Red) and total cellular ROS (DCFH) in HeLa with or without FX11. Cells were treated with 1 µM rotenone, antimycin, or oligomycin in serum-free RPMI-1640 medium for 2 h and loaded with DCFH-DA or MitoSOX Red with or without 15 µM FX11 in the same medium containing ETC inhibitors. Fluorescence signals were captured by confocal microscope imaging. **b** Total DCFH fluorescence signal of single cells from experiment (**a**) were calculated for analysis. **c** Total MitoSOX Red fluorescence signal of single cells from experiment (**a**) were calculated for analysis. **d** Antimycin showed concentration-dependent induction of mitochondrial ROS/superoxide (MitoSOX Red) and total cellular ROS (DCFH) in HeLa with or without FX11. **e** Total DCFH fluorescence signal of single cells from experiment (**d**) were calculated for analysis. The blue and red areas represent the conversion from dominant antioxidative to dominant prooxidant activity of LDH or vice versa. The intersection point represents equal antioxidative and pro-oxidative activity of LDH. **f** Total MitoSOX Red fluorescence signal of single cells from experiment (**d**) were calculated for analysis. **g** The statistical data of the mitochondrial ETC modulators did not significantly change total cellular ROS (DCFH) in HeLa/ρ0 with or without FX11. **h** The statistical data of the mitochondrial ETC modulators did not significantly change mitochondrial ROS/superoxide (MitoSOX™ Red) in HeLa/ρ0 with or without FX11. **i** The statistical data of a serial concentration of antimycin showed no induction of total cellular ROS (DCFH) in HeLa with or without FX11. **j** The statistical data of a serial concentration of antimycin showed no induction of mitochondrial ROS/superoxide (MitoSOX™ Red) in HeLa cells. Data were confirmed by at least three independent experiments and expressed as mean ± SEM. The experimental details are described in Materials and Methods

These agents increased mitochondrial ROS/superoxide and total cellular ROS (Fig. 4a, first and third panels; Fig. 4b–c). In the presence of FX11, mitochondrial superoxide level with or without treatment of rotenone, antimycin, or oligomycin was comparable with each other (Fig. 4a, c). FX11 treatment increased total cellular ROS level in the control cells but reduced ROS in the treated cells to a similar level (Fig. 4a, forth panel; b). The results suggest that without agents interfering with ETC, LDHs inhibit ROS production, agreeable with previous reports^27,28,32^; with agents interfering with ETC, LDH enhances ROS production, consistent with our observations on the in vitro enzyme assays (Fig. 1a–f), H_2_O_2_-producing activity of LDH enhanced by mitochondria (Fig. 1g, j, k), H_2_O_2_ production by cells with or without LDH knockout (Fig. 2c, i), and HeLa/ρ0 cells (Fig. 3).

The above results suggest that the amount of superoxide/ROS generated from mitochondria is a key to regulate cytosolic LDH between its antioxidative activity and pro-oxidative activity in cells, or the antioxidative and pro-oxidative activity of LDH co-played with mitochondria to regulate ROS level in cancer cells. To further demonstrate this, we treated cells with serial concentrations of antimycin, which induced a dose-dependent increase of mitochondrial superoxide and cellular ROS (Fig. 4d upper and middle panels, Fig. 4e, f). When LDH was inhibited by FX11, cellular ROS was brought to a similar level (Fig. 4d bottom panel), which is independent of the antimycin concentrations—the strength of the mitochondrial oxidative stress. The antimycin dose-dependent curves of cellular ROS with or without FX11 inhibition intersected at one point (Fig. 4e), where the blue and red area indicated the dominant antioxidative activities and the dominant pro-oxidative activities of LDH, respectively, and the intersection point reflected the equal anti- and pro-oxidative activities of LDH under the experimental setting (Fig. 4e).

We further used HeLa/ρ0 cells to perform the same experiments. Since ETC was defective in ρ0 cells, these agents had no significant effect on MitoSOX RED signal but a moderate effect on DCFH signal (Fig. 4g–h). In the presence of FX11, DCFH signal reduced significantly (Fig. 4g, i). Neither DCFH nor MitoSOX RED signal was relevant with antimycin concentration, and in the presence of FX11, DCFH signal reduced to a similar level (Fig. 4i–j). The results also support that amplification of ROS by LDH requires mitochondria with functional ETC.

Together, the results suggested that LDH in mitochondria-intact cancer cells could switch between antioxidative and pro-oxidative activity, depending on the strength of oxidative stimuli.

### The effect of LDH knockout on ETC modulator- or hypoxia-induced cellular ROS

Next, we used LDH-knockout cells to study the relationship between LDH activity and cellular ROS levels and the results (Supplementary Fig. 8a, b) were consistent with the data in Fig. 3 and the previous report.^27^ A little surprise is that LDHA or LDHB knockout did not change the level of mitochondrial ROS level probed by MitoSOX^TM^ Red (Supplementary Fig. 8b). However, as this is not the focus of this study, we did not further pursue the molecular mechanism.

**Fig. 5:**
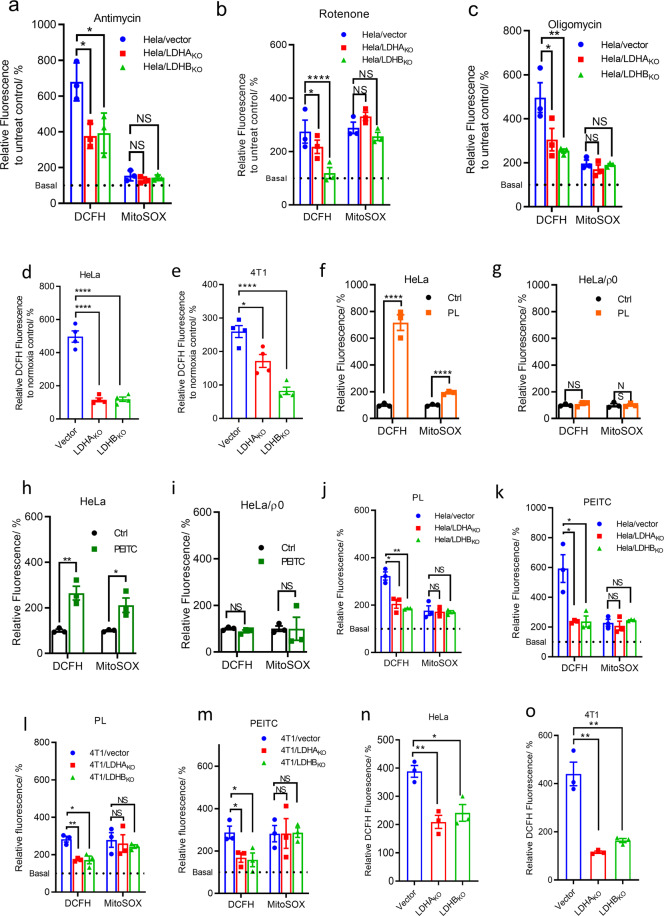
The relationship between LDH knockout and cellular ROS in HeLa and 4T1 cells. Induction of ROS in mitochondrial (MitoSOX™ Red) and total cellular level (DCFH) in LDHA or LDHB knockout cells by antimycin (**a**), rotenone (**b**), and oligomycin (**c**). Cells were treated with 1 µM rotenone, antimycin, or oligomycin in serum-free RPMI-1640 medium for 2 h and loaded with DCFH-DA or MitoSOX Red in the same medium containing ETC inhibitors/uncoupler. Hypoxia-induced ROS (DCFH) change in HeLa/vector, HeLa/LDHA_KO_, HeLa/LDHB_KO_ cells (**d**) and 4T1/vector, 4T1/LDHA_KO_, 4T1/LDHB_KO_ cells (**e**). PL-induced mitochondrial ROS/superoxide (MitoSOX™ Red) and total cellular ROS (DCFH) in HeLa (**f**) and HeLa/ρ0 cells (**g**). PEITC-induced mitochondrial ROS/superoxide (MitoSOX™ Red) and total cellular ROS (DCFH) in HeLa (**h**) and HeLa/ρ0 cells (**i**). **j** PL-induced mitochondrial ROS/superoxide and cellular ROS in vector control HeLa/vector, HeLa/LDHA_KO_, and HeLa/LDHB_KO_ cells. The fluorescence is relative to untreated cells respectively, which is 100% and showed as dash lines. **k** PEITC-induced mitochondrial ROS/superoxide and cellular ROS in vector control HeLa/vector, HeLa/LDHA_KO_, and HeLa/LDHB_KO_ cells. The fluorescence is relative to untreated cells respectively, which is 100% and showed as dash lines. **l** PL-induced mitochondrial ROS/superoxide and cellular ROS in 4T1/vector, 4T1/LDHA_KO_, and 4T1/LDHB_KO_ cells. The fluorescence is relative to untreated cells, which is 100% and shown as dash lines. **m** PEITC-induced mitochondrial ROS/superoxide and cellular ROS in 4T1/vector, 4T1/LDHA_KO_, and 4T1/LDHB_KO_ cells. The fluorescence is relative to untreated cells, which is 100% and shown as dash lines. Doxorubicin-induced ROS (DCFH) in HeLa/vector, HeLa/LDHA_KO_ and HeLa/LDHB_KO_ cells (**n**), and in 4T1/vector, 4T1/LDHA_KO_, and 4T1/LDHB_KO_ cells (**o**). Fluorescence signals were captured by confocal microscope imaging. ROS fluorescence intensity was normalized to their untreated counterparts (ROS level of untreated cells were designated 100%). Data were confirmed by at least three independent experiments and expressed as Mean ± SEM. The experimental details are described in Materials and Methods

Rotenone, antimycin, and oligomycin all increased mitochondrial ROS/superoxide in HeLa/vector, HeLa/LDHA_KO_ and HeLa/LDHB_KO_ cells in a similar degree (Fig. 5a–c), but induced a significantly higher fold change of cellular ROS in control cells than in HeLa/LDHA_KO_ and HeLa/LDHB_KO_ cells (Fig. 5a–c). We then used 4T1/vector, 4T1/LDHA_KO_ and 4T1/LDHB_KO_ cells to repeat above experiments and obtained similar results (Supplementary Fig. 8c, Supplementary Fig. 9). Hypoxia could increase superoxide production in complex III in ETC.^33^ Hypoxia (1% oxygen) induced less ROS increase (DCFH signal) in HeLa or 4T1 LDH knockout cells than in their vector controls, comparing with DCFH signal in those cells under normoxia (20% oxygen) (Fig. 5b). Taken together, ETC modulators and hypoxia enhanced production of mitochondrial ROS/superoxide and cellular ROS, LDHA or LDHB knockout selectively attenuated cytosolic ROS production, indicating that LDHA and LDHB were responsible for cytosolic ROS amplification.

### The relevance of LDH with anticancer agent-induced ROS

Piperlogumine (PL)^34^ and phenethyl isothiocyanate (PEITC)^35^ are typical anticancer agents that kill cancer cells. The mechanism by which these agents to induce ROS is partly through depleting cellular glutathione.^34,35^ On the other hands, we previously noticed that PEITC and PL could also enhance mitochondrial ROS production,^36^ which let us propose that PL- or PEITC-induced ROS production may involve two phases, initiation and amplification. In the initiation phase, PEITC or PL enhance superoxide/ROS production in mitochondria, in the amplification phase, superoxide/ROS released from mitochondria initiates LDH to amplify H_2_O_2_/ROS. To support this notion, we provided following evidence.

PL or PEITC induced a significant elevation of mitochondrial ROS/superoxide and total cellular ROS in HeLa cells, but not in HeLa/ρ0 cells (Fig. 5f–i); using another pair of cells, HCT116 and HCT116/ρ0 cells, we obtained the same results (Supplementary Fig. 10 a–d), indicating that PL or PEITC-induced ROS requires mitochondria with intact ETC.

We then treated HeLa/vector cells, HeLa/LDHA_KO_, and HeLa/LDHB_KO_ cells with PL or PEITC. While HeLa/vector cells showed a significant increase of both cellular ROS and mitochondrial ROS/superoxide, HeLa/LDHA_KO_, and HeLa/LDHB_KO_ cells exhibited an increase of mitochondrial ROS/superoxide comparable to HeLa/vector cells but a significantly smaller increase of cellular ROS (Fig. 5j, k); we then treated 4T1/vector, 4T1/LDHA_KO_, and 4T1/LDHB_KO_ cells with PL or PEITC, and obtained the same results (Fig. 5l, m). The results demonstrated that LDH knockout selectively inhibited cellular ROS induced by PL or PEITC without affecting mitochondrial ROS/superoxide induced by PL or PEITC.

Therefore, the PEITC- or PL-induced ROS could be dissected into two parts, mitochondrial and cytosolic ROS. Because LDHA or LDHB knockout only selectively inhibited PEITIC- or PL-induced cytosolic ROS but not mitochondrial ROS, LDH is responsible for amplification of cytosolic ROS. Hence, the results support the notion that PEITC- and PL-enhanced ROS production involves ROS initiation in mitochondria and LDH-mediated amplification of cytosolic ROS.

Besides PL and PEITC, doxorubicin (DOX) enhances ROS production through generating ROS/superoxide via quinone one-electron redox cycling.^37^ We tested if LDH was involved in the amplification of doxorubicin-induced ROS. We treated wild-type cells (HeLa/vector and 4T1/vector) and LDHA or LDHB knockout cells with the drug and observed LDHA or LDHB knockout significantly attenuate DOX-induced ROS (Fig. 5n, o).

In order to confirm if LDH amplification of ROS induced by PL, PEITC, or DOX is a general phenomenon in cancer cells, we treated 8 cell lines (Supplementary Fig. 10e, f, g) with these drugs with or without FX11. The results showed that FX11 significantly inhibited or even abolished the ROS production induced by these drugs.

We treated cells with or without LDH knockout cells with PL, PEITC, or DOX, there were no significant difference of cell survival (Supplementary Fig. 11). This was consistent with our previous study.^36^ We previously investigated the quantitative relationship between cell death and ROS induced by PL, PEITC, or DOX, and our results demonstrated that cell death was dissociated from the ROS amount induced by these agents.

### The effect of LDH knockout on intra-tumor oxidative stress

The above experiments demonstrated that LDH could exhibit either antioxidative or pro-oxidative activity in cancer cells, which depends on the experimental conditions (Figs. 1, 3, 4). The next question is which activity of LDH, pro-oxidative or antioxidative, is dominant in cancer cells in vivo.

**Fig. 6:**
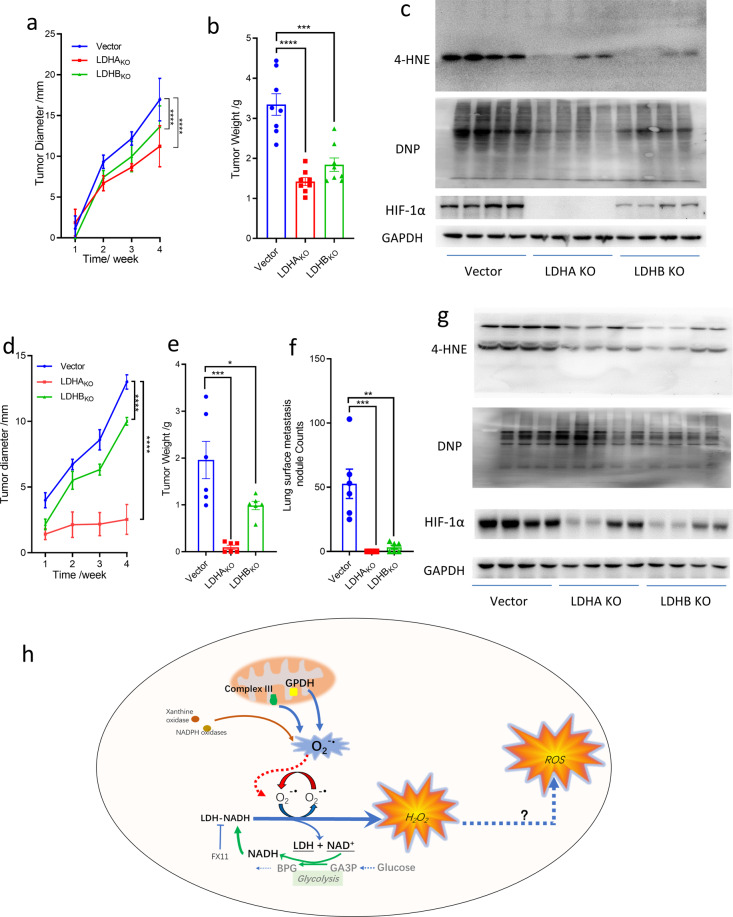
LDH knockout inhibits tumor growth and metastasis and reduces oxidative stress. LDH-knockout inhibits HeLa tumor growth (**a**) and reduces tumor weight (**b**). **c** 4-HNE-modified protein, total protein carbonyl groups and HIF-1α expression of the tumor homogenates prepared from the tumors formed by HeLa/vector, HeLa/LDHA_KO_, HeLa/LDHB_KO_ cells, respectively. **d**, **e** LDH-knockout inhibits 4T1 tumor growth and reduces tumor weight. **f** Lung surface metastasis of 4T1/vector, 4T1/LDHA_KO_, 4T1/LDHB_KO_ cells, counted under dissection microscope. **g** 4-HNE-modified protein, total protein carbonyl groups and HIF-1α expression of the tumor homogenates prepared from the tumors formed by 4T1/vector, 4T1/LDHA_KO_, 4T1/LDHB_KO_ cells, respectively. Scale bar, 1 cm. **h** Oxidative stimuli (rotenone, antimycin, oligomycin, PL, PEITC) cause an increase of electron leakage from ETC in the inner membrane of mitochondria and the electron is captured by molecular oxygen to produce superoxide. Specifically, superoxide formed in the complex III and mitochondrial glycerol-3-phosphate dehydrogenase (mGPDH) on the inner membrane of mitochondria can be released toward the intermembrane space or cytosol side.^7,8^ The mitochondrial outer membrane is permeable to superoxide, which then diffuses from intermembrane space to cytosol^9^ and serves as an initiator to trigger a free radical chain reaction on LDH-bound NADH, leading to amplification of total cellular ROS. Alternatively, other sources of superoxide, such as superoxide produced from quinone one-electron cycle,^37^ NADPH oxidase,^10^ xanthine oxidase,^10^ etc., can also serve as an initiator to trigger LDH-mediated ROS amplification. Glycolysis continuously provides hydride to reduce NAD^+^ to NADH at the step of GAPDH, linking glycolysis with ROS production via LDH. Data were expressed as Mean ± SEM. GA3P glyceraldehyde-3 phosphate, BPG 1,3-bisphosphateglyerate, GAPDH glyceraldehyde-3 phosphate dehydrogenase

We inoculated HeLa LDH-knockout cells subcutaneously in NOD/SCID female mice. LDH knockout significantly reduced tumor growth (Fig. 6a, b). Moreover, we inoculated 4T1 LDH-knockout cells in the mammary fat pad of BALB/c mice. The 4T1 cells are highly tumorigenic and invasive. Inoculation of 4T1 cells in mammary fat pad of BALB/c mice produced primary tumors and could spontaneously metastasize to multiple distant organs, with the disease progression similar to human breast cancer.^38^ LDHA and LDHB knockout significantly reduced the growth rate of tumors and lung metastasis with the LDHA knockout more prominent (Fig. 6d–f).

To investigate the oxidative stress in these tumors, we used 4-HNE (4-Hydroxynonenal) and protein carbonylation as the biomarkers.^39,40^ Oxidative stress induced by H_2_O_2_ could result in accumulation of 4-HNE protein modification.^39^ Protein carbonyl groups are often introduced by various oxidative modifications at side chains,^40^ and can be conjugated by 2,4-Dinitrophenol (DNP). We observed that LDHA and LDHB knockout both decreased 4-HNE and protein carbonylation level in tumor xenografts (HeLa, Fig. 6c; 4T1, Fig. 6g) indicating that knockout LDHA or LDHB decreases the oxidative stress in tumor.

As ROS stabilizes hypoxia-inducible factor-1 alpha (HIF-1α) and promotes tumor progression,^41,42^ we determined if LDHA or LDHB knockout, which reduces oxidative stress, could also reduce HIF-1α protein level in tumors. The result showed a significant decrease of HIF-1α in tumors formed by LDH-knockout cells (Fig. 6c, g). Collectively, LDHA and LDHB knockout significantly reduces oxidative stress in tumors formed by Hela and 4T1.

## Discussion

Increased H_2_O_2_ production is a characteristics of cancer cells,^1,2^ but the underlying mechanism is not completely understood. According to current understanding, H_2_O_2_ production in cancer cells involves two steps: mitochondrial ETC and enzymes likes NADPH oxidases generate superoxide radical, then mitochondrial SODII and cytosolic SODI catalyzes the disproportionation of superoxide, converting two molecules of superoxide to one molecule of H_2_O_2_ and one molecule of molecular oxygen. In this study, we propose that LDH could also contribute to H_2_O_2_ production in cancer cells. In theory, superoxide in cells should initiate LDH to amplify H_2_O_2_ production the same as the superoxide in aqueous solution to initiate LDH to amplify H_2_O_2_ production, as outlined in the reaction scheme in the section of introduction.^5^ Here, we showed that LDH contribute significantly to H_2_O_2_ production in cancer cells. LDHA or LDHB knockout dramatically reduced H_2_O_2_ production in Hela and 4T1 cells. Re-expression of LDHA in Hela/LDHA_KO_ cells partially restored H_2_O_2_ production. The data are somehow surprising, because the data suggested that a large portion of total H_2_O_2_ in Hela and 4T1 cells is derived from LDH-mediated reactions (Fig. 2).

In line with previous work,^3–5^ we may propose a novel mechanism of H_2_O_2_ production in cancer cells (Fig. 6h) comprising of two phases: the first phase is generation of superoxide, which could be derived from mitochondria,^7,8,24^ or from other sources, such as superoxide from quinone one-electron redox cycling,^37^ NADPH oxidase,^10^ xanthine oxidase^10^ etc.; in the second phase, superoxide initiates LDH to catalyze NADH oxidation and produce H_2_O_2_, following the reaction scheme in the section of Introduction.^5^ The high glycolysis rate efficiently recycles NAD back to NADH to support H_2_O_2_ generation by LDH. In cancer cells, in the presence of ferrous ion as a catalyst, H_2_O_2_ is converted to hydroxyl free radical, which is readily reactive with cellular components on its path. H_2_O_2_ could also react with nitrite to form nitrogen dioxide free radical.^43^ However, the biochemical process from H_2_O_2_ to ROS amplification in this model requires further investigation.

LDHA are overexpressed in many tumors^14–16^ and LDHB is overexpressed in some cancers such as human lung adenocarcinoma with KRAS mutation and testicular germ cell tumors.^44–46^ High LDHA is associated with a poor prognosis,^14–16^ but the mechanism is unknown. H_2_O_2_ production by LDH may provide a clue to it, as H_2_O_2_ can act as a signaling molecule and play important roles in cancer initiation and development including mutation, cell proliferation, angiogenesis, and metastasis.^47^

In cancer cells, LDH can exhibit antioxidative and pro-oxidative activities simultaneously. While the pro-oxidative activity is dependent on its enzyme property, the antioxidative activity is independent of the enzyme property but dependent on the altered mitochondrial metabolism induced by perturbation of LDH.^27^ Under certain conditions, the antioxidative activity of LDH dominates, while under other conditions, the pro-oxidative activity of LDH dominates (Figs. 1, 3, 4, Supplementary Figs. 1, 2, 8, 10). It should be noted that the dominance of one activity only means that this activity is larger than the other one under such condition and the dominance of one activity over another is condition dependent. This suggests that LDH plays an important role in regulating cellular redox state by its bifunctional activities.

To specify the differences between the pro-oxidative and antioxidative activity of LDH, we did the following analysis. We demonstrated that knockout of LDHA or LDHB significantly reduced H_2_O_2_ production by Hela or 4T1 cells, and re-expression of LDHA in Hela/LDHA_KO_ virtually restored H_2_O_2_ production (Fig. 2). This provides a direct link between H_2_O_2_ production and LDH in cancer cells, which is supported by enzymological data (Figs. 1 and 2) and the well-established theoretical basis by previous studies.^3–5^ On the contrary, LDH knockdown or knockout increased DCFH signal (Fig. 3e). Although DCFH signal is reflecting an increase of total ROS in cells, DCFH is a poor probe for H_2_O_2_, as it has very low rate constant to react with H_2_O_2_.^43^ These data could to some extent specify the differences between the pro-oxidative and antioxidative activity of LDH.

Although perturbation of LDH on cellular ROS production by cancer cells in vitro has been previously documented,^27^ the effect of perturbation of LDH on the oxidative stress in vivo is unknown. Our study indicated that in tumor xenograft models, LDHA and LDHB knockout both significantly decreased the oxidative stress in the tumors scored by the biomarkers 4-HNE and protein carbonylation, indicating that LDH’s pro-oxidative activity is higher than its antioxidative activity in tumors. In addition, LDHA and LDHB knockout both significantly reduced HIF-1α level. As ROS could stabilize HIF-1α,^41,42^ it might act as a link between LDH and HIF-1α.

Finally, our study provides a mechanistic understanding of a link between LDH and anticancer agents that enhance ROS production in cancer cells. Induction of ROS in cancer cells is conceived as a promising pharmacological approach to treat cancers.^34,35^ Because cancer cells have a higher basal ROS level than normal cells,^1,48^ proper dosing of ROS-inducers may increase the ROS to a lethal level in cancer cells but a sublethal level in normal cells, hence selectively kill cancer cells. There are many anticancer agents, which kill cancer cells mainly or partly via induction of cellular ROS.^34,35^ Regarding the mechanism of ROS induction by pharmacological agents, previous studies mainly focus on perturbation of the redox balance between cellular antioxidant and oxidant system.^34,35^ In this study, we propose that the model compounds PEITC and PL induce ROS production via two phases: they enhance superoxide production in mitochondria, then the superoxide released to cytosol is used as initiator to amplify LDH-mediated ROS production (Fig. 6h).

## Materials and methods

### Measurement of H_2_O_2_-producing activity of LDH in cell free system

The principle to measure H_2_O_2_ generation by LDH is based on following rationales. First, superoxide and hydroperoxyl radicals are in equilibrium in aqueous solution with pKa = 4.8^4^ and superoxide can serve as initiator for LDH to generate H_2_O_2_ through a free radical chain reaction.^5^ Second, the H_2_O_2_ can be detected by Amplex^TM^ Red-HRP system as previously described,^49^ wherein the horseradish peroxide (HRP) catalyzed oxidation of fluorogenic probe Amplex^TM^ Red by H_2_O_2_ with a 1:1 stoichiometry ratio. Hence, we set a coupled enzyme assay by linking LDH with AmplexTM Red-HRP system and this coupled enzyme assay could measure the rate of LDH-generated H_2_O_2_. All the reactions were performed in 50 mM Tris Buffer containing 5 μM NADH, 0.02% BSA, 50 μM Amplex^TM^ Red, 1 U/mL horseradish peroxidase, pH7.4 (note, 5 μM NADH is the optimized concentration for this assay). The appropriate amount of LDHA or LDHB (100 U/mL or other indicated concentration) was added at last to initiate the reaction. After thorough mixing, 150 µL mixture of each sample was loaded to 96-well black plate. The emission fluorescence at 585 ± 15 nm with the excitation wavelength 525 ± 15 nm was recorded by SpectraMAX i3 Multi-Mode microplate reader (Molecular Devices) for 10 min linearly. The fluorescence signals from samples without LDH were subtracted from the readings as background correction. For the inhibition assay, the LDH inhibitors FX11 and oxamate were added to the reaction mixture at the indicated concentration. Bovine LDH from heart (LDHB) and from muscle (LDHA) were purchased from Sigma-Aldrich.

### Measurement of the enhancement of H_2_O_2_-producing activity of LDH by mitochondria

According to the previously reported method,^25,26^ in a reaction system containing mitochondria, succinate, and rotenone, a fraction of electron would leak from ETC and captured by molecular oxygen to form superoxide. The superoxide released to mitochondrial matrix side is then converted to hydrogen peroxide by intramitochondrial SOD. On the other hand, superoxide released to intermembrane side may be used as an initiator for LDH to generate hydrogen peroxide. We used the previously reported method,^25,26^ and we added LDH and NADH into the reaction system. Briefly, the reaction mixture contains MiR05 buffer, 10 mM succinate, 5 μM rotenone, 200 μg-protein/mL isolated mitochondria, 5 μM NADH, 50 μM Amplex^TM^ Red, 1 U/mL horseradish peroxidase, pH7.4, in a total volume of 100 μl/well in a 96-well black plate. The reaction was recorded at the emission fluorescence at 585 ± 15 nm with the excitation wavelength 525 ± 15 nm for 10 min by a SpectraMAX i3 Multi-Mode microplate reader (Molecular Devices) for 10 min. To test GPDH produced ROS, succinate and rotenone were replaced by 1 mM glycerol-3-phosphate and others remained the same.

### Measurement of H_2_O_2_ excretion rate of intact cells

Cells were plated in 96-well plate overnight, and was washed three times by pre-warmed Hank’s Balanced Salt Solution (HBSS). 100 µL detection buffer with 1 U/mL HRP and 50 µM AmplexRed in pre-warmed HBSS was added. The reaction was recorded at the emission fluorescence at 585 ± 15 nm with the excitation wavelength 525 ± 15 nm for 10 min by a SpectraMAX i3 Multi-Mode microplate reader (Molecular Devices).

### Microscopy imaging of intracellular ROS

ROS measurement is probed by 2′,7′-dichlorodihydrofluorescein diacetate (DCFH-DA, Sigma), MitoSOX^TM^ Red mitochondrial superoxide indicator (Invitrogen), according to manufacturers’ instruction. Cells were pre-incubated with or without PL (10 µM for 3.5 h), PEITC (10 µM for 3.5 h), doxorubicin (10 µg/mL for 3.5 h), rotenone (1 µM for 1.5 h), oligomycin (1 µM for 1.5 h), antimycin (1 µM for 1.5 h), EUK134 (20 µM for 1.5 h), or mitoTEMPO (20 µM for 1.5 h) in serum-free RPMI-1640 medium. After incubation, FX11 was added to medium to achieve 15 µM final concentration and cells were incubated for another 0.5 h. For DCFH-DA staining, 10 µM final concentration of DCFH-DA was added at the same time points as FX11, i.e. 30-min loading; for MitoSOX^TM^ Red, 2.5 µM final concentration was added at the last 10 min of the second FX11 incubation. Stained cells were washed with ice cold HBSS (Hank’s Balanced Salt Solution, pH 7.2), then observed under a Zeiss LSM710 laser confocal microscope (Carl Zeiss, Germany) equipped with Zen software to process the image. The intensity of fluorescence was analyzed by ImageJ software.

### Treatment of cancer cells with PL, PEITC, DOX and FX11

For cells treated with PL and PEITC, cells were cultured in medium containing 10 µM of PL or PEITC for 4 h, as described.^35,50^ For cells treated with doxorubicin, cells were cultured in medium containing 10 µg/ml doxorubicin for 4 h. For cells treated with FX11, cells were cultured in medium containing 15 µM of FX11 for 30 min.

### Clone and expression of LDH proteins

Total mRNA was extracted from HeLa cell using RNeasy Mini Kit (Qiagen) according to manufacturer’s protocol. The LDHA and LDHB genes were firstly amplified with first round of PCR from total mRNA, then the second round of PCR was used to amplify the coding domain sequence of enzymes with the following primers: LDH-A-1st-F: 5′-AGCTGTTCCACTTAAGGCCC-3′, LDH-A-1st-R: 5′-GGGTTGCCCAAGAATAGCCT-3′, LDH-A-2nd-F: 5′-ggaattcCATATGGCAACTCTAAAGGATCAG-3′, LDH-A-2nd-R: 5′-ataagaatGCGGCCGCATGATATGACATCAGAAGACTT-3′, LDH-B-1st-F: 5′-TCCAGAGCCTTCTCTCTCCT-3′, LDH-B-1st-R: 5′-GGCTTTGATTCTGTGAGCCC-3′, LDH-B-2nd-F: 5′-ggaattcCATATGGCAACTCTTAAGGAAAAACTC-3′, LDH-B-2nd-R: 5′-ataagaatGCGGCCGCAGAGCTCACTAGTCACAGGT-3′. The final PCR products were cloned into pET28 a (+) expression plasmid. Then the protein-coding plasmids were transferred into BL21 (DE3) *Escherichia* *coli* by standard transformation protocol. Bacterium pelleted from 250 mL LB culture broth were lysed in PBS with 10 mM PMSF (Thermo Scientific) by sonication. After 16,000 *g* and 10 min’ centrifugation, the supernatant was collected for enzyme purification.

### LDH proteins purification

The crude LDH proteins were first extracted by ammonium sulfate precipitation. Ammonium sulfate was added to protein supernatant slowly with constant stirring on ice. The supernatant from 40% saturation ammonium sulfate solution was precipitated again by adding ammonium sulfate to 60% saturation. After centrifugation, protein pellet was dissolved in PBS with 0.5 M NaCl and 10 mM PMSF. The crude protein solution was kept at 4 °C and purified by Ni-Sepharose affinity chromatography later. The Ni-Sepharose column (Sangon Biotech, Shanghai) was first washed and equilibrated by 10 mM imidazole (Sigma) with 0.5 M NaCl (pH 8.8). Then the protein solution was loaded into the column. After 20 column volumes’ wash by washing buffer (100 mM imidazole with 0.5 M NaCl, pH 8.8), the LDH protein was eluted by 10 column volume of elution buffer (300 mM imidazole with 0.5 M NaCl, pH 8.8). The eluents were concentrated by ultrafiltration using centrifugal filters with cut-off value 10-kD (Millipore).

### Mitochondria extraction

Cell mitochondria were extracted by pre-cooled extraction buffer 1 (300 mM Sucrose, 10 mM HEPES, 0.2 mM EDTA, pH adjusted to 7.4. 0.1% BSA was added before use). About 10^8^ cells were pelleted and homogenized in 2 mL extraction buffer with Dounce homogenizer. The homogenate was centrifuged at 600 *g* for 5 min, and the supernatant was further centrifuged at 10,000 *g* for 10 min. The final precipitate containing purified mitochondria was re-suspended in MiR05 buffer (110 mM Sucrose, 60 mM K-lactobionate, 20 mM HEPES, 10 mM KH_2_PO_4_, 3 mM MgCl_2_, 0.5 mM EGTA, 0.1% fatty acid-free BSA, pH 7.1.) and kept on ice.^51^

Tissue mitochondria were extracted by pre-cooled extraction buffer 2 (67 mM sucrose, 50 mM Tris, 50 mM KCl, 10 mM EDTA, pH 7.4. 0.1% BSA was added before use). Tissues (mouse muscle, xenograft or patient tumor) were minced with scissors and then digested in 10 volumes of 0.05% trypsin with 10 mM EDTA for 20 min on ice. Samples were centrifuged at 600 *g* for 10 min after digestion. The pellet was re-suspended in 10 volumes of extraction buffer 2 and homogenized by Dounce homogenizer. Mitochondria was enriched by differential centrifugation and kept in MiR05 buffer on ice as mentioned above.

### Cell and mitochondria oxygen consumption rate analysis

Oxygen consumption rate was analyzed on Oxygraph-2K platform (Oroboros Instruments Corp, Austria) according to manufacturer’s protocol. 10^6^/mL Cells in RPMI-1640 medium or 25 μg/mL protein of mitochondria in MiR05 buffer were injected into assay chamber. For cell assay, oligomycin (1 μM), rotenone (1 μM), FCCP (1 μM), antimycin (1 μM) were sequentially injected into the assay chambers when the oxygen consumption rate was stable. For mitochondria assay, succinate (10 mM), ADP (5 mM) and oligomycin (2.5 μM) were sequentially injected into the assay chambers when the oxygen consumption rate was stable. The soluble oxygen concentration was recorded for data analysis.

### Animal study

HeLa/vector HeLa/LDH_KO_ and HeLa/LDHB_KO_ cells (10^6^ cells per mouse) were subcutaneously inoculated in Nude/SCID mice and LDH knockout 4T1/vector, 4T1/LDH_KO_ and 4T1/LDHB_KO_ cells (10^5^ cells per mouse) were subcutaneously inoculated in BALB/C mice. Tumor size was monitored every week. The tumors were collected 4 weeks after inoculation. Tumors were weighted and then homogenized for western blot analysis of 4-HNE modification and total protein carbonyl group. Lung surface metastasis of 4T1 tumor was also counted under dissection microscope. For mitochondria extraction, 10^6^ HeLa cells or 10^5^ 4T1 cells were subcutaneously inoculated in Nude/SCID mice or BALB/C mice, respectively, and tumors were collected 3 weeks after inoculation and used for mitochondria isolation. The animal protocols for animal experiments were approved by the Committee of Animal Experimental Centre at the Zhejiang Chinese Medical University.

### Patient samples

Cholangiocarcinoma samples were collected when surgery was necessary to remove the tumor burden. All sample collection procedures were reviewed and approved by the ethics committee of the Second Affiliated Hospital Zhejiang University School of Medicine.

### Statistics

All data were analyzed using the GraphPad Prism software (GraphPad Prism 7.0). Tumor growth curve was analyzed by using multivariant statistical analysis. Two-tailed Student’s *t*-test was used for other statistical comparison, and significance was defined at *p < 0.05, **p < 0.01, ***p < 0.001, ****p < 0.0001.

## Supplementary information

Supplementary information_revised

## Data Availability

The data sets used for the current study are available from the corresponding author upon reasonable request.
