# Supplementary material for: Lactate dehydrogenases amplify reactive oxygen species in cancer cells in response to oxidative stimuli
Source: Signal Transduct Target Ther. 2021 Jun 28;6:242. doi: 10.1038/s41392-021-00595-3 (PMC8236487; doi:10.1038/s41392-021-00595-3)
Supplement: Supplementary file 1 — Supplementary information_revised [file 41392_2021_595_MOESM1_ESM.pdf]

Supplementary Materials for

Lactate dehydrogenases amplify reactive oxygen species in cancer cells in response to oxidative stimuli

Hao Wu[1#], Yuqi Wang[1#], Minfeng Ying[1], Chengmeng Jin[1], Jiangtao Li[2], Xun Hu[1*]

[1]Cancer Institute (Key Laboratory for Cancer Intervention and Prevention, China National Ministry of Education, Zhejiang Provincial Key Laboratory of Molecular Biology in Medical Sciences), The Second Affiliated Hospital, Zhejiang University School of Medicine, China

[2]Department of Surgery, The Second Affiliated Hospital, Zhejiang University School of Medicine, China

[#] These authors contributed equally.

* Correspondence: Xun Hu, Ph.D., 88 Jiefang Road, Hangzhou, China, Email: huxun@zju.edu.cn

This PDF file includes:

Materials and methods

Supplementary figure 1 -11

Materials and methods

Cancer cell lines. Human cervical cancer HeLa cells, human breast cancer Bcap37 cells, mouse breast cancer 4T1 cells, human gastric cancer MGC803 cells were maintained in complete RPMI-1640 medium (Gibco) with 10% FBS and 2 mM L-glutamine. Human colorectal carcinoma HCT116 were maintained in complete McCoy's 5A medium (Gibco) with 10% FBS and 2 mM L-glutamine. Human liver cancer HepG2 cells were maintained in complete MEM medium (Gibco) with 10% FBS and 2 mM L-glutamine. Human lung cancer A549 cells were maintained in complete F-12K medium (Gibco) with 10% FBS and 2 mM L-glutamine. Human pancreatic cancer Mia cells were maintained in complete DMEM medium (Gibco) with 10% FBS and 2 mM L-glutamine. The ρ0 cells were maintained in corresponding complete medium with 50 ng/mL Ethidium bromide (EB), 2 mM pyruvate, and 50 μg/mL uridine. All the medium contained 100 U/ml penicillin/streptomycin. HeLa cells were incubated in hypoxic incubator of 1% oxygen, 5% carbon dioxide, 94% nitrogen for hypoxia incubation assay. The incubation time was 6 hours.

Reagents. Glutamine, pyruvate, oxamate, ethidium bromide, uridine, dichloro-dihydro-fluorescein diacetate, PL, PEITC and doxorubicin were purchased from Sigma-aldrich. Penicillin/streptomycin solution, Amplex^{TM} Red and MitoSOX^{TM} Red were purchased from ThermoFisher Scientific, Inc. LDH inhibitor FX11 was from Calbiochem. LDH from bovine heart (sigma, L2625), bovine muscle (sigma, L1378) and rabbit muscle (sigma, L2500) were purchased from Sigma-Aldrich.

Mitochondrial DNA-depleted ρ_0 cell induction and characterization. HeLa cell and HCT116 cell were seeded into 6-well plate at density of 100 cells /well. 50 ng/mL Ethidium bromide (EB), 2 mM pyruvate, 50 μg/mL uridine were added to culture medium after overnight incubation. The cells

were sub-cultured in the same condition when necessary. One and half months later, cells were collected, and total DNA was extracted by Blood & Cell Culture DNA Mini Kit (Qiagen). The mitochondrial DNA encoded genes were amplified with the following primers: ND1-F: 5’-CAACATCGAATACGCCGCAG-3’, ND1-R: 5’-AATCGGGGGTATGCTGTTCG-3’, ND2-F: 5’-AACCCTCGTTCCACAGAAGC-3’, ND2-R: 5’-AGCTTGTTTCAGGTGCGAGA-3’, ND3-F: 5’-CCGCGTCCCTTTCTCCATAA-3’, ND3-R: 5’-GGCCAGACTTAGGGCTAGGA-3’, ND4L-F: 5’-TCGCTCACACCTCATATCCTC-3’, ND4L-R: 5’-AGGCGGCAAAGACTAGTATGG-3’, ND4-F: 5’-TTTCCTCCGACCCCCTAACA-3’, ND4-R: 5’-CGTAGGCAGATGGAGCTTGT-3’, ND5-F: 5’-GCCCAATTAGGTCTCCACCC-3’, ND5-R: 5’-GCAGGAATGCTAGGTGTGGT-3’, ND6-F: 5’-ACCCACAGCACCAATCCTAC-3’, ND6-R: 5’-GATTGTTAGCGGTGTGGTCG-3’, COX1-F: 5’-CTTTTCACCGTAGGTGGCCT-3’, COX1-R: 5’-GGCGTAGGTTTGGTCTAGGG-3’, COX2-F: 5’-GCTGTCCCCACATTAGGCTT-3’, COX2-R: 5’-GCTCTAGAGGGGGTAGAGGG-3’, COX3-F: 5’-AGGCATCACCCCGCTAAATC-3’, COX3-R: 5’-CCGTAGATGCCGTCGGAAAT-3’, CYTB-F: 5’-TCTTGCACGAAACGGGATCA-3’, CYTB-R: 5’-GTGGGGAGGGGTGTTTAAGG-3’. The PCR products were then subjected to gel electrophoresis analysis.

Transient siRNA knockdown of LDH. The following siRNAs targeting LDH were synthesized LDH-A: 5’-GGCAAAGACTATAATGTAA-3’, LDH-B: 5’-GGGAAAGTCTCTGGCTGAT-3’, NC: 5’-GATCATACGTGCGATCAGA-3’. The siRNA was transfer to HeLa cells using RNAiMAX reagent (Invitrogen) according to manufacturer’s protocol. Briefly, after HeLa cell reached 80% confluence in 6-well plate, the siRNA and the RNAi reagent were mixed with Opti-MEM medium and added to plate drop by drop. The cells were subjected to fluorescent microscopy analysis for ROS or collected for LDH activity or western blot analysis 96 hours after transfection.

Gene knock-out using CRISPR/Cas9. For generation of the CRISPR/Cas9 knockout cell line, gRNA-expressing plasmids were constructed using the px459 vector. Cells were transiently transfected with the appropriate plasmid by Lipofectamine 3000 reagent (Invitrogen) according to the manufacturer's instructions and screened in the presence of puromycin (2.5 μg/mL for HeLa cells and 3.5 μg /mL for 4T1 cells). Single knockout clones were verified by Western Blot and sequencing of the PCR fragment. The following gRNA sequence was used for HeLa cells LDHA/B knockout: LDHA-F: 5'-CACCGTCATCGAAGACAAATTGAA-3', LDHA-R: 5'-CACCGTCATCGAAGACAAATTGAA-3', LDHB-F:5'- CACCACTTGCTCTTGTGGATGTTT-3', LDHB-R: 5'- AAACAAACATCCACAAGAGCAAGT-3'. The following gRNA sequence was used for 4T1 cells LDHA/B knockout: LDHA-F: 5'-CACCGCTGGTCATTATCACCGCGG-3', LDHA-R: 5'- AAACCCGCGGTGATAATGACCAGC-3', LDHB-F: 5'-CACCGACGGCAGGAGTCCGCCAGC-3', LDHB-R: 5'- AAACGCTGGCGGACTCCTGCCGTC-3'.

LDHA re-expression in HeLa cells. LDHA re-expression in HeLa cells. The pLJM1-LDHA vector or blank vector were transfer to HeLa/$LDHA_{KO}$ cells using RNAiMAX reagent (Invitrogen) according to manufacturer's protocol. The cells were subjected to fluorescent microscopy analysis for ROS or collected for LDH activity or western blot analysis 48 hours after transfection.

Western Blot Analysis. Cells were lysed with M-PER mammalian protein extraction reagent (Pierce, USA), supplemented with protease inhibitor cocktail (Thermo Scientific, USA). Protein concentration was measured by BCA protein assay (Thermo Scientific, USA). After heat denaturation, samples were stored at -80 °C before use. The protein was applied to a 10% to 12% SDS polyacrylamide gel, transferred to a PVDF membrane, and then detected by the proper primary and secondary antibodies before visualization by Western Lighting Plus ECL kit (Perkin Elmer, USA).

The primary antibodies used: rabbit anti-LDHA (Cell Signaling Technology, USA), rabbit anti-LDHA (Santa Cruz Biotechnology, USA), mouse anti-4 Hydroxynonenal (Abcam,USA).

Total protein carbonyl group assay. Tumor xenografts were dissected, weighted and flash frozen in liquid nitrogen immediately. After ground into fine powder, tumors tissues were further homogenized with M-PER mammalian protein extraction reagent (Thermo Scientific, USA), supplemented with protease inhibitor cocktail. Protein carbonyl groups were assayed as described previously[1] with some modification. Briefly, denatured protein extracts were separated by SDS-PAGE and blotted on PVDF membrane and washed by 2N HCl for 5 minutes, followed by 10 minutes staining in 0.5mM 2,4-dinitrophenylhydrazine (DNPH)/2N HCl solution. The membrane was sequentially washed by 2N HCl 5 times and methanol 7 times. The washed membrane was then blocked with 5% milk and immunostained with anti-DNP antibody as conventional Western Blotting procedures.

LDH enzyme activity assay. Cells maintained in complete growth medium at 70% confluence was washed by PBS and lysed by M-PER buffer (Thermo Fisher Scientific). The supernatants after centrifugation (14,000 rpm/30 min/ 4°C) were collected for further analysis. Supernatants or purified enzymes were added to reaction buffer (50 mM Tris-HCl, 1 mM pyruvate, 100 µM NADH, pH 7.4) to initiate reaction. The absorbance at 340 nm wavelength was recorded at 25°C with a spectrophotometer. For the K_i and V_{max} value calculation, 15µM FX11 (Calbiochem) was added to reaction buffer and the initial velocities under difference concentrations of NADH were recorded.

Cell viability. LDH knockout HeLa or 4T1 cells were seeded at the density of 3000 cells/well in 96-well plate. Series concentration of PEITC, PL, doxorubicin(DOX) were added the second day and

cells were incubated for another 72 hours. Cell viability was assayed by CellTiter 96® AQueous One Solution Cell Proliferation Assay kit (Promega).

References:

[1] Levine, R. L., Williams, J. A., Stadtman, E. R. & Shacter, E., Carbonyl assays for determination of oxidatively modified proteins. *Methods Enzymol* **233** 346 (1994).

Supplementary figures and figure legends

Supplementary Fig 1 related to Fig 1

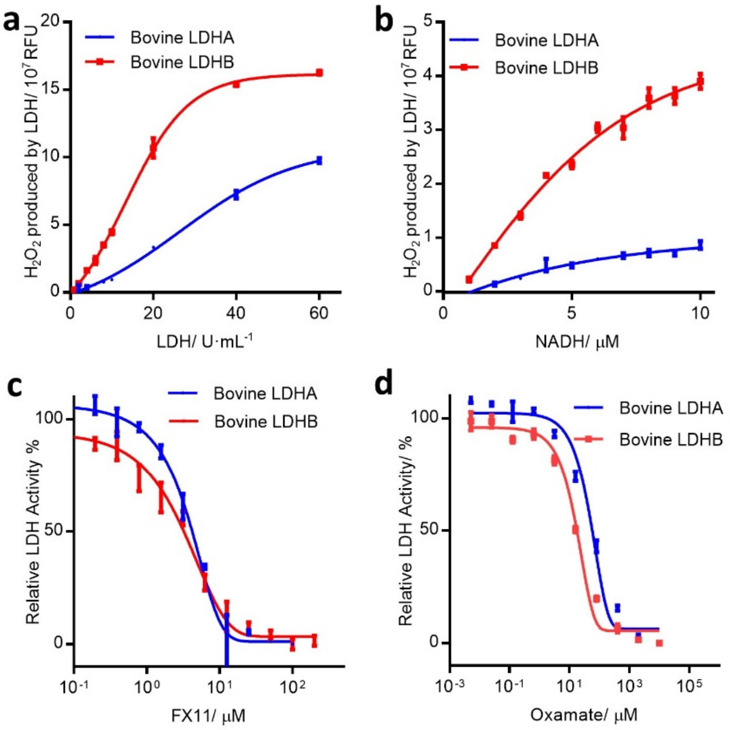

e

	Km (μM, NADH)	Vmax (μmol/mg protein/min)	Ki (μM)
Human LDHA	6.2 ± 0.7	114.9 ± 3.6	10.1 ± 2.1
Human LDHB	3.6 ± 0.9	95.5 ± 3.3	13.6 ± 7.5
Bovine LDHA	5.6 ± 0.8	118.1 ± 3.0	12.7 ± 3.5
Bovine LDHB	7.5 ± 0.4	113.6 ± 1.1	82.1 ± 30.6

Supplementary Fig 1. The H_2O_2-generating activity of bovine LDHA and B. LDH from bovine muscle (LDHA dominant) and bovine heart (LDHB dominant) were incubated with NADH and the H_2O_2 generation was detected by Amplex Red-HRP system, as described in Methods. LDH inhibitors were added together as indicated. **(a)** LDH activity-dependent H_2O_2 generation. 1 unit of bovine LDH refers the conversion of 1 μmole of pyruvate into lactate in 1 minute. **(b)** NADH concentration-dependent H_2O_2 generation by bovine LDH. **(c)** FX11 inhibits bovine LDH-catalyzed H_2O_2 generation. **(d)** Oxamate inhibits bovine LDH-catalyzed H_2O_2 generation. **(e)** The kinetic parameters of human and bovine LDH in converting pyruvate to lactate and K_i refers the LDH inhibitor FX11. Experiments were repeated 3 times and one representative data is showed and expressed as mean ± SD. n = 3. The experimental details are described in Materials and Method.

Supplementary Fig 2 related to Fig 1

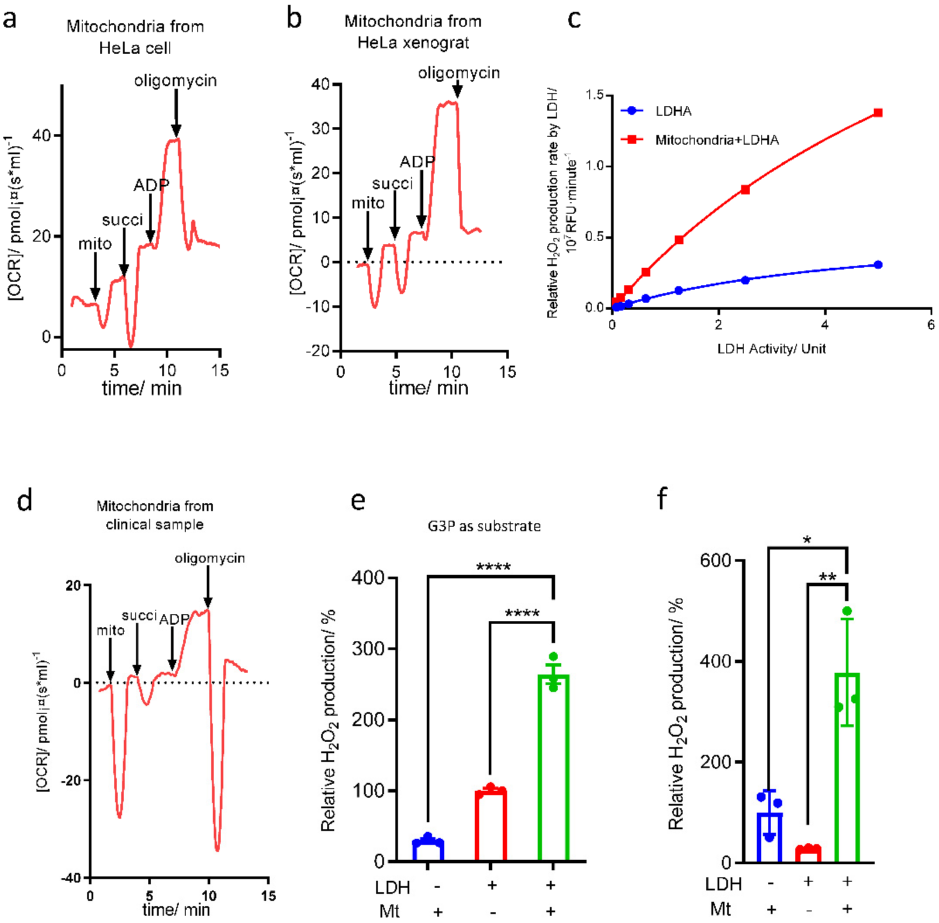

Supplementary Fig 2. Mitochondria activity. (a) Respiration assay of isolated mitochondria from HeLa cell line showed the mitochondria were functional. **(b)** Respiration assay of isolated mitochondria from HeLa cell-derived xenografts. **(c)** Mitochondria prepared from HeLa cell-derived xenografts enhanced the H_2O_2-generation activity of recombinant human LDHA in enzyme concentration-dependent manner. Succinate and rotenone were added to stimulate mitochondrial ROS production. **(d)** Respiration assay of isolated mitochondria from patient's tumor. **(e)** Glycerol 3-phosphate (G3P) stimulated ROS also enhanced the H_2O_2-generation activity of recombinant human LDHA. **(f)** Mitochondria from cultured HeLa cell line significantly increased the H_2O_2-generation activity of rabbit muscle LDH. Succinate and rotenone were added to stimulate mitochondrial ROS production. Experiments were repeated 3 times and one representative data is showed and expressed as mean ± SD. n = 3. The experimental details are described in Materials and Methods. Mt, mitochondria.

Supplementary Fig 3 related to Fig 1

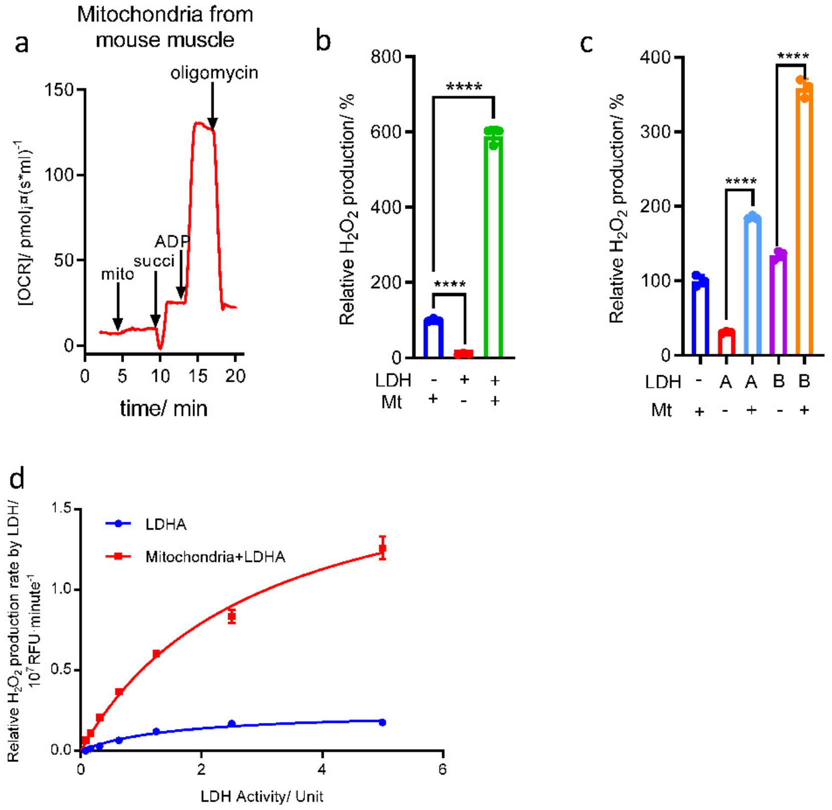

Supplementary Fig 3. (a) Respiration assay of isolated mitochondria from BALB/C mice muscle showed the mitochondria were functional (Oxygen consumption rate (OCR). **(b)** Mitochondria from Mice muscle enhanced rabbit muscle type LDH generated H_2O_2. **(c)** Mitochondria prepared from mice muscle enhanced the H_2O_2 generation activity of recombinant human LDH. **(d)** Mitochondria prepared from mice muscle enhanced the H_2O_2 generation activity of recombinant human LDHA in enzyme concentration dependent manner. Experiments were repeated 3 times and one representative data is showed and expressed as mean ± SD. n = 3. The experimental details are described in Materials and Methods. Mt, mitochondria.

Supplementary Fig 4 related to Fig 2

Sequencing verification of gene knockout

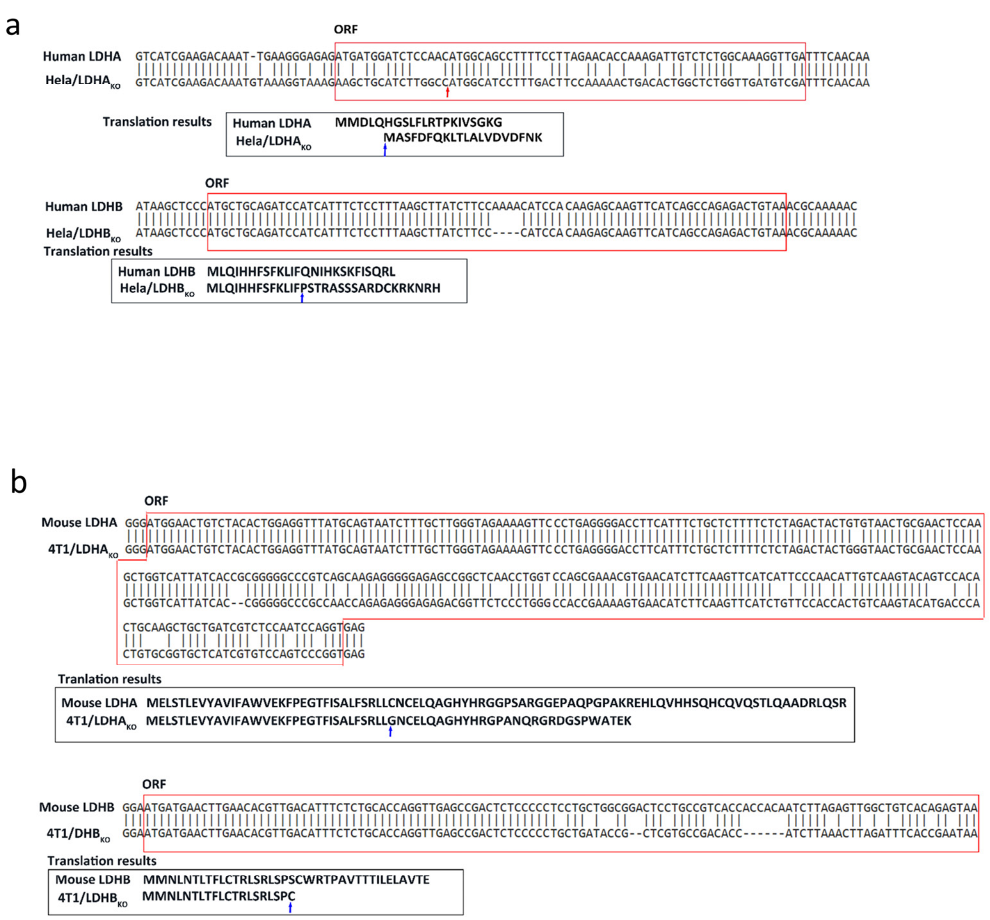

Supplementary Fig 4. The mutation spectra of target gene in knockout cell lines. The original gene sequences and each mutant clone were showed in alignment. The red frames showed ORF (Open Reading Frame， ORF) around the target sequences. Between original sequences and mutant clones, the different bases mean point mutant and short lines mean base deletion or insert. The translation results showed protein sequences translated from the ORF respectively. The red arrow showed the altered start codon location of modified genes and the blue arrows showed the first amino acid that different from original peptide. **(a)** HeLa/LDHA$_{KO}$ and HeLa/LDHB$_{KO}$ clone cells aligned with human-LDHA, -LDHB original sequences for ORF gene and proteins. **(b)** 4T1/LDHA$_{KO}$ and 4T1/LDHB$_{KO}$ clone cells aligned with mouse-LDHA, -LDHB original sequences for ORF gene and proteins.

Supplementary Fig 5 related to Fig 3

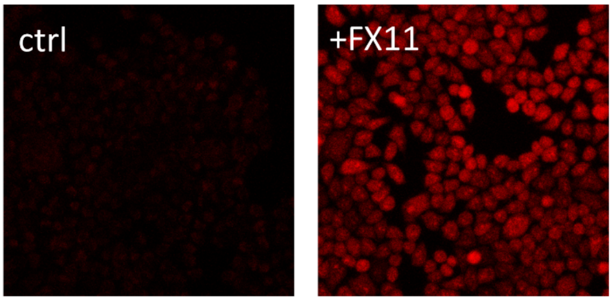

Supplementary Fig 5. LDH inhibitor FX11 increased mitochondrial ROS in HeLa cells. HeLa cells were treated with or without 15µM FX11 in serum-free RPMI-1640 medium for 30 minutes. At the last 10 minutes, cells were loaded with MitoSOX Red probe in the same medium containing FX11 and then washed by ice-cold HBSS buffer 3 times and imaged by confocal microscope.

Supplementary Fig 6 related to Fig 3
Mitochondria ROS scanvenger

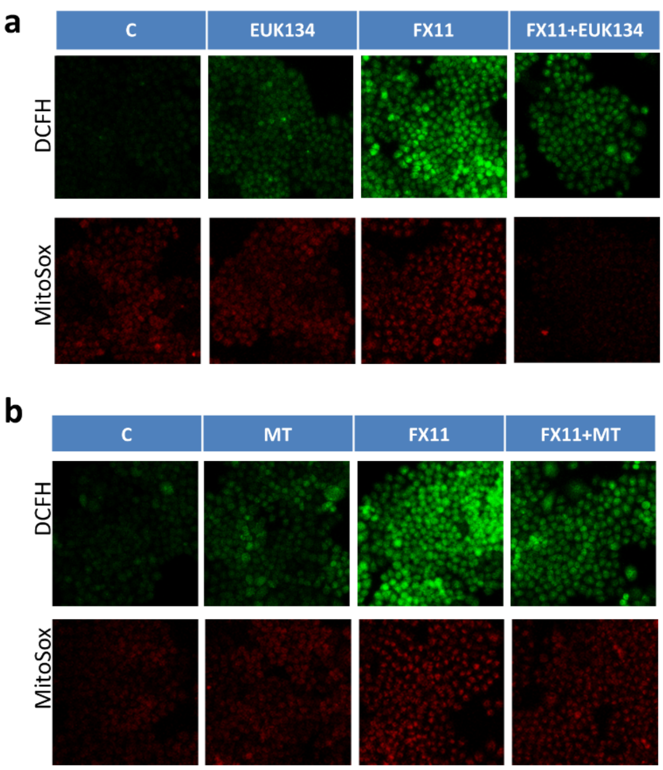

Supplementary Fig 6. Mitochondria ROS scavenger decreased FX11 induced ROS in Hela cells. HeLa cells were pre-treated with mitochondrial ROS scavengers EUK134 and mitoTEMPO (MT) in serum-free RPMI-1640 medium for 1.5 hours, and then loaded with DCFH-DA or MitoSOX Red probe in the same medium containing scavengers, with or without FX11. Cells were washed by pre-cooled HBSS buffer for 3 times and imaged under confocal microscope. **(a)** EUK134 decreased FX11 induced ROS in Hela cells. **(b)** mitoTEMPO (MT) decreased FX11 induced ROS in Hela cells.

Supplementary Fig 7 related to Fig 3

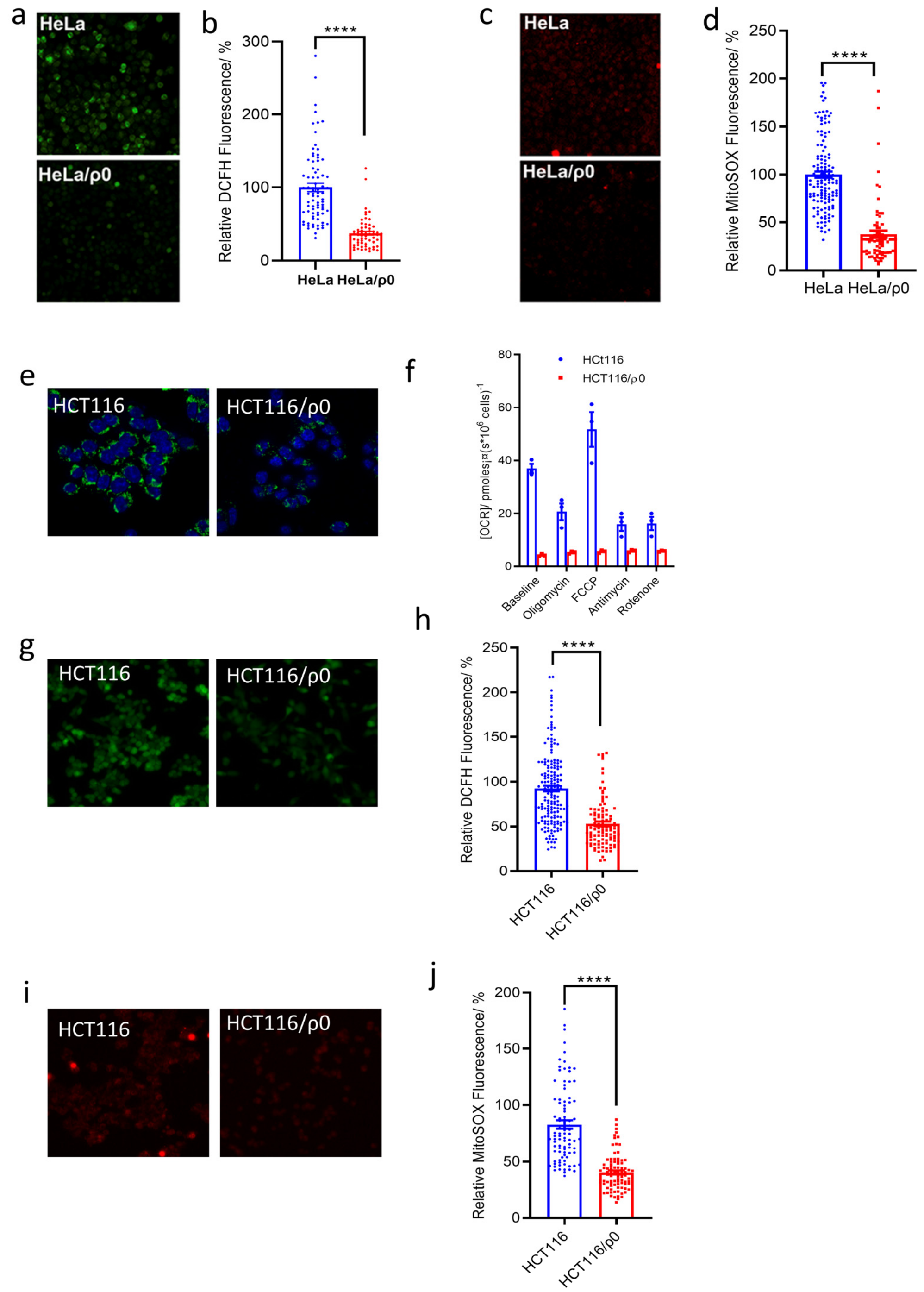

Supplementary Fig 7. Characterization of HeLa/ρ0 and HCT116/ρ0 cells. **(a-b)** HeLa/ρ0 cells demonstrate lower basal total cellular ROS level than HeLa cells. **(c-d)** HeLa/ρ0 cells demonstrate lower basal mitochondrial ROS (superoxide) level than HeLa cells. **(e)** MitoTraker Green staining of HCT116 and HCT116/ρ0 cells show a less mitochondria mass in HCT116/ρ0 cells. **(f)** Oxygen consumption rate (OCR) in comparison to HCT116 cells; **(g-h)** DCFH signal in comparison to HCT116 cells; **(i-j)** MitoSOX™ Red signal in comparison to HCT116 cells. Data were confirmed by at least 2 independent experiments. For experimental details, see Materials and Methods.

Supplementary Fig 8 related to Fig 5

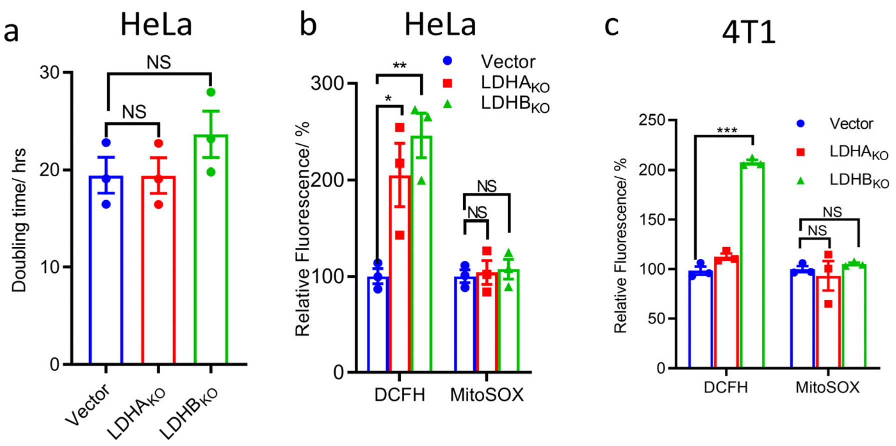

Supplementary Fig 8. The relationship between cellular ROS and LDH knockout in 4T1 cells. **(a)** Doubling time of HeLa/vector, HeLa/LDHA$_{KO}$, and HeLa/LDHB$_{KO}$. **(b)** Relative total cellular ROS level probed by DCFH and mitochondrial ROS probed by MitoSOX™ Red of HeLa/vector, HeLa/LDHA$_{KO}$, and HeLa/LDHB$_{KO}$. **(c)** Relative total cellular ROS level probed by DCFH and mitochondrial ROS probed by MitoSOX™ Red of 4T1/vector, 4T1/LDHA$_{KO}$, and 4T1/LDHB$_{KO}$. Data were confirmed by at least 2 independent experiments. The experimental details are described in Materials and Methods.

Supplementary Fig 9 related to Fig 5

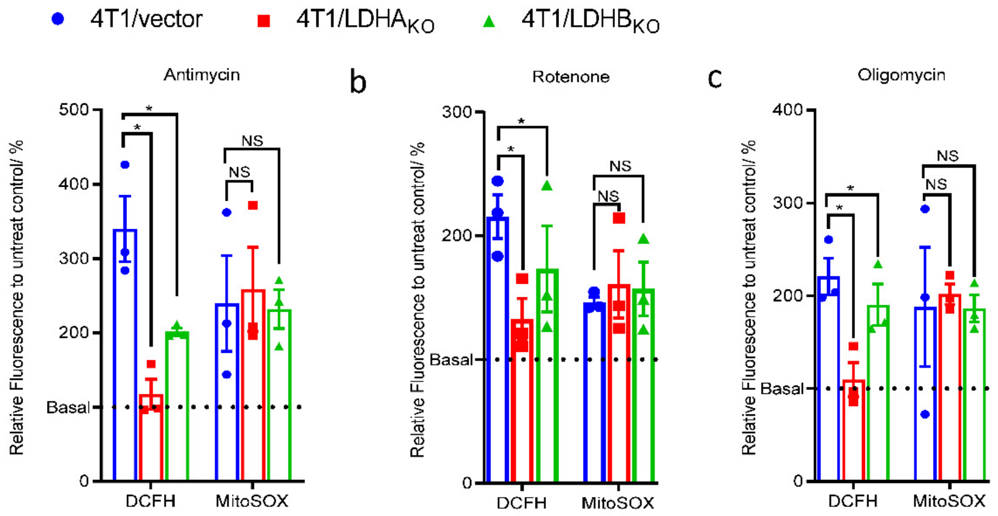

Supplementary Fig 9. The relationship between LDH knockout and cellular ROS in 4T1 cells. (a-c) Induction of ROS in mitochondrial (MitoSOX™ Red) and total cellular level (DCFH) in LDHA or LDHB knockout cells by antimycin **(a)**, rotenone **(b)**, and oligomycin **(c)**. Cells were treated with 1μM rotenone, antimycin, or oligomycin in serum-free RPMI-1640 medium for 2 hours and loaded with DCFH-DA or MitoSOX Red in the same medium containing ETC inhibitors.

Supplementary Fig 10 related to Fig 5

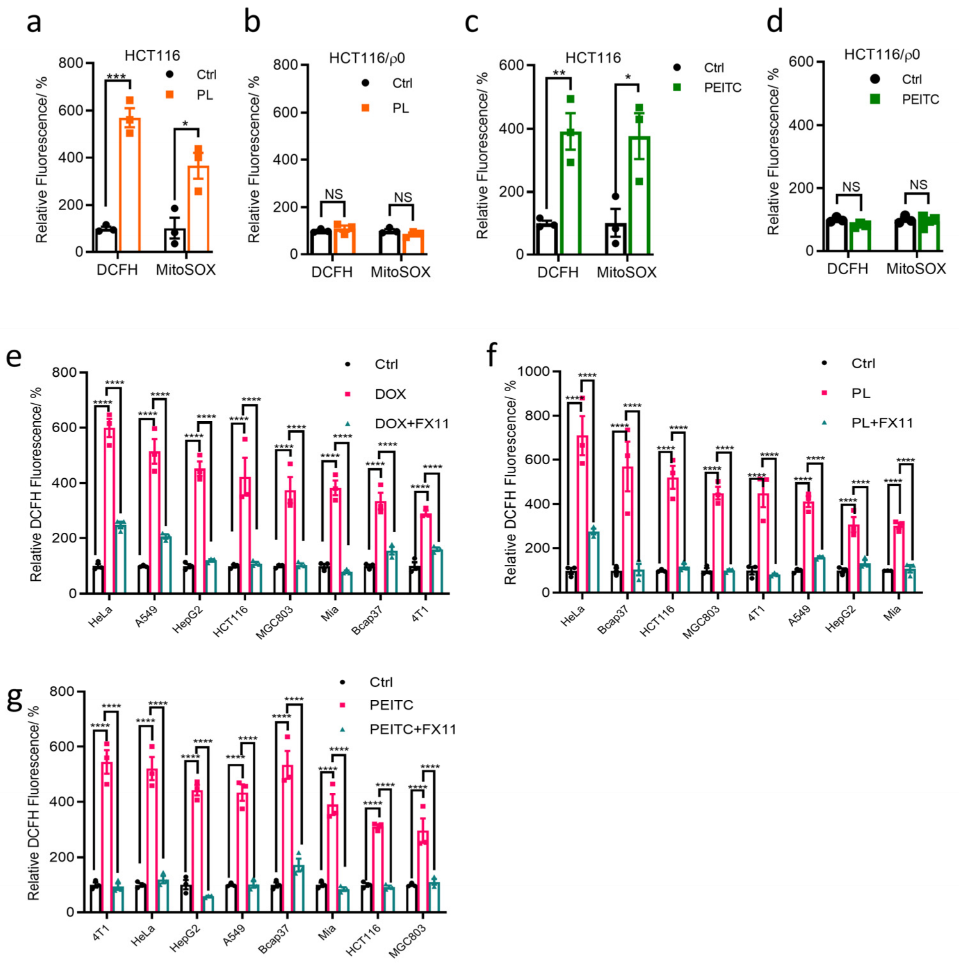

Supplementary Fig 10. (a&b) PL-induced mitochondrial ROS/superoxide (MitoSOX™ Red) and total cellular ROS (DCFH) in HCT116 and HCT116/ρ0 cells. **(c&d)** PEITC-induced mitochondrial ROS/superoxide (MitoSOX™ Red) and total cellular ROS (DCFH) in HCT116 and HCT116/ρ0 cells. **(e-g)** Doxorubicin-, PL-, or PEITC- induced ROS (DCFH) in 8 cell lines with or without FX11. Data were confirmed by at least 2 independent experiments. The experimental details are described in Materials and Methods.

Supplementary Fig 11 related to Fig 5

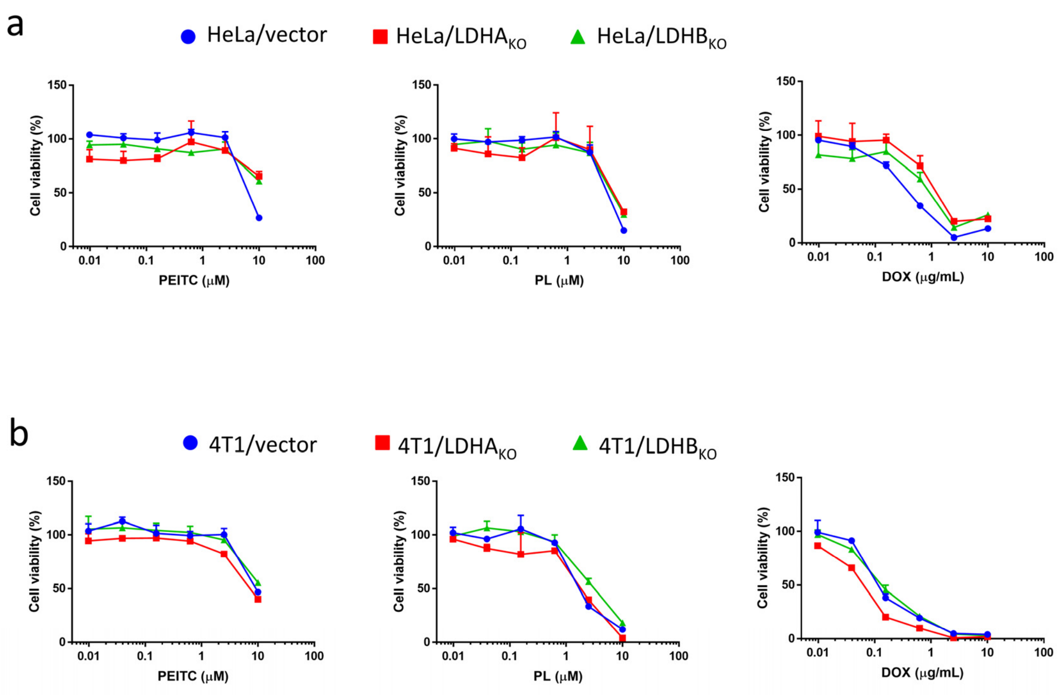

Supplementary Fig 11. The drug sensitivity of LDH knockout cells. LDH knockout HeLa cells or 4T1 cells were exposed series concentration of PEITC, PL, or doxorubicin (DOX) for 72 hours. The viable cells were assayed by CellTiter 96® AQueous One Solution Cell Proliferation Assay kit and normalized to control cells.
